# Differential Gene Expression Analysis in *Polygonum minus* Leaf upon 24 h of Methyl Jasmonate Elicitation

**DOI:** 10.3389/fpls.2017.00109

**Published:** 2017-02-06

**Authors:** Reyhaneh Rahnamaie-Tajadod, Kok-Keong Loke, Hoe-Han Goh, Normah M. Noor

**Affiliations:** Institute of Systems Biology (INBIOSIS), Universiti Kebangsaan MalaysiaBangi, Malaysia

**Keywords:** differentially expressed gene, *de novo* transcriptome, Illumina sequencing, elicitation, *Persicaria minor*

## Abstract

*Polygonum minus* is an herbal plant that grows in Southeast Asian countries and traditionally used as medicine. This plant produces diverse secondary metabolites such as phenolic compounds and their derivatives, which are known to have roles in plant abiotic and biotic stress responses. Methyl jasmonate (MeJA) is a plant signaling molecule that triggers transcriptional reprogramming in secondary metabolism and activation of defense responses against many biotic and abiotic stresses. However, the effect of MeJA elicitation on the genome-wide expression profile in the leaf tissue of *P. minus* has not been well-studied due to the limited genetic information. Hence, we performed Illumina paired-end RNA-seq for *de novo* reconstruction of *P. minus* leaf transcriptome to identify differentially expressed genes (DEGs) in response to MeJA elicitation. A total of 182,111 unique transcripts (UTs) were obtained by *de novo* assembly of 191.57 million paired-end clean reads using Trinity analysis pipeline. A total of 2374 UTs were identified to be significantly up-/down-regulated 24 h after MeJA treatment. These UTs comprising many genes related to plant secondary metabolite biosynthesis, defense and stress responses. To validate our sequencing results, we analyzed the expression of 21 selected DEGs by quantitative real-time PCR and found a good correlation between the two analyses. The single time-point analysis in this work not only provides a useful genomic resource for *P. minus* but also gives insights on molecular mechanisms of stress responses in *P. minus*.

## Introduction

Due to the sessile lifestyle, plants have to cope with different changes of biotic/abiotic factors in their surrounding environments to survive in nature. However, this defense response is costly and often setback by the repression of growth (Yang et al., 2012b). In response to stresses, plants convert resource allocation from growth to the biosynthesis of defensive compounds which is energetically demanding (Attaran et al., 2014). Plants produce a wide range of secondary metabolites for coordinated plant response to various internal and external cues (Davies and Schwinn, 2003). Plant secondary metabolites comprise a large group of lipophilic volatile organic compounds (VOCs) with high vapor pressure and low molecular weight. VOCs serve as signal molecules to mediate plant communication with their environment for herbivore deterrence, attraction of pollinators, seed dispersers, and protection against different stresses (Dudareva et al., 2013).

Beside their main role in defending plants from environmental stresses, investigations on the biosynthesis of VOCs have also attracted much interest for their applications as pharmaceuticals, pesticides, fragrance, and flavoring (Crozier et al., 2008). However, due to low abundance of these secondary metabolites (<1% dry weight) and high industrial demands, some plants in the wild which produce valuable compounds are overharvested and exposed to endangerment (Yang et al., 2014). Therefore, a comprehensive knowledge of metabolic pathways and regulatory mechanisms involved in the biosynthesis of VOCs is necessary to understand their role in defense responses and to enhance their yields for various applications.

Increased accumulation of volatile secondary metabolites in plants leads to enhanced stress tolerance and the activation of defense mechanisms (Aranega-Bou et al., 2014). A multilevel network of biosynthesis pathways and regulation of secondary metabolites converges on complex hormone signaling cascades in which jasmonates (JAs) play a crucial role in transcriptional control of plant defense gene expression and metabolism (Attaran et al., 2014).

JAs consisting jasmonic acid (JA) and its cyclopentanon derivatives such as its volatile methyl ester (methyl jasmonate, MeJA), are plant-specific endogenous signaling phytohormones that have long been observed to be potent regulators of elicitor signals for the biosynthesis of plant secondary metabolites (Zhao et al., 2005). This endogenous hormone regulates a variety of physiological plant processes such as root elongation, vegetative growth, production of viable pollen, senescence, cell cycle regulation, fruit ripening, production of specialized metabolites, plant response to wounding and defenses against pathogens, insects, and notorious pests (Farmer et al., 2003). It is well-studied that elicitation is a common method of inducing the accumulation of plant defensive secondary metabolites in whole plant or cell culture (Memelink et al., 2001). Previous genome-wide transcript profiling studies revealed that the addition of JA elicitors such as MeJA induces an extensive transcriptional reprogramming (De Geyter et al., 2012) leading to activation of several metabolic pathways including terpenoids (Misra et al., 2014), phenylpropanoids (Cocetta et al., 2015), and alkaloids (Kang et al., 2004). In addition to defense induction, the exogenous MeJA elicitation affects other plant functions including ontogeny, vegetative growth, and photosynthesis (Moreira et al., 2009). Previous studies showed that exogenous application of MeJA induces defense responses and reduces growth in several species (Heijari et al., 2005; Nabity et al., 2012; Yang et al., 2012b; Noir et al., 2013; Attaran et al., 2014).

*Polygonum minus* Huds (syn. *Persicaria minor*), commonly known as “kesum” in Malaysia, is an aromatic herbal plant in the Polygonaceae family which is widely used as a flavoring ingredient in local cuisine “asam laksa” and traditionally used to treat digestive disorders (Christapher et al., 2015). *P. minus* leaf is rich in VOCs comprising terpenoids (Baharum et al., 2010), aldehydes (Yaacob, 1990), and phenolic compounds (Maizura et al., 2011). *P. minus* leaf extracts possess high free radical scavenging activity and reducing power due to the high content of total phenolic compounds (Qader et al., 2011). This plant was shown to have antiviral, antimicrobial and anticancer activity (Vikram et al., 2014). Hence, it has a great potential for various applications in the pharmaceutical and perfumery industries for its essential oil.

Previously, an elicitation experiment using MeJA was performed on *P. minus* leaves through cDNA-amplified fragment length polymorphism (AFLP) transcript profiling approach with reported effects on some of the genes involved in secondary metabolite biosynthesis (Ee et al., 2013). However, detailed understanding of the biological mechanism of MeJA-mediated production of secondary metabolites and whole transcriptome changes was incomplete due to limitations in the gel-based selection of cDNA-AFLP transcripts for sequencing.

To date, there are only 3352 expressed sequence tags (ESTs) of *P. minus* available in the NCBI database from previous study (Roslan et al., 2012). In the current study, we aim to identify differentially expressed genes (DEGs) in the MeJA-elicited *P. minus* leaf at 24 h compared with mock treatment. To gain an in-depth knowledge of gene expression changes in MeJA-elicited leaves, RNA-seq was employed to greatly expand the genomic resources (Loke et al., 2016). Over 192 million short pair-end reads were generated by Illumina HiSeq™ 2000 platform and used for *de novo* transcriptome assembly through Trinity analysis pipeline. The assembled UTs were analyzed to identify DEGs for functional annotation and downstream analyses. This is the first report on genome-wide transcriptional response in *P. minus* leaf elicited by MeJA with a focus on the biosynthesis of secondary metabolites. Furthermore, understanding the molecular mechanisms underlying MeJA elicitation will assist in genetic engineering of targeted secondary metabolites in *P. minus*.

## Materials and methods

### Plant materials and MeJA treatment

*P. minus* stem cuttings were collected from Genting Highland (3° 25′ 42.18″ N, 101° 47′ 21.45″ E) and propagated in controlled environment chambers (A1000, Conviron, Canada) at 22/16°C day/night temperatures under 12 h light/dark photoperiod with light intensity of 170 ± 20 μmol m^−2^ s^−1^ at ~75% RH. After 45 days (Khairudin et al., 2014), an aqueous solution of 150 μM MeJA (Sigma-Aldrich) (Ismail et al., 2011) and 0.01% (v/v) Tween 20 was sprayed on all plant leaves to the point of runoff. The control (mock-treated) plant leaves were sprayed with only 0.01% (v/v) Tween 20. After elicitation, treated and control plants were placed separately in different growth chambers. Expanded young leaves from apical parts of plants (Ee et al., 2013) were harvested at 24 h after treatment and immediately frozen in liquid nitrogen before storage at −80°C. Two biological replicates from independent control and treated plants were prepared for RNA sequencing. qRT-PCR was performed using three biological and three technical replicates.

### RNA isolation and cDNA library construction

Total RNA was extracted from 1 g of control and MeJA-treated leaf samples using a modified Lopez-Gomez method (Lopez-Gomez and Gomez-Lim, 1992) by adding 50% PVP-40 due to high phenolic compounds and polysaccharides in *P. minus*. RNA purity was quantified by A260:A280 ratios using ND-1000 Nanodrop spectrophotometer (Thermo Scientific) and the quality of RNA was visualized through agarose gel electrophoresis. RNA integrity numbers (RIN) of RNA samples for RNA-seq were determined by Agilent 2100 Bioanalyzer. RNA samples with RIN > 7 were sent to BGI (Shenzhen, China) for cDNA library preparation and RNA sequencing. Briefly, transcriptome sequencing of four cDNA paired-end libraries were constructed using TrueSeq RNA kit (Illumina) following the manufacturer's protocols, starting with 4 μg of total RNA.

### RNA sequencing and assembly

Approximately 13.9 Gb of 192.17 million 90 bp paired-end reads were generated on Illumina HiSeq™ 2000 platform. The raw sequence data of each sample condition (Treated and Control) which associated with this study were deposited in GenBank under BioProject accession numbers PRJNA279327 and PRJNA208436 respectively. The raw reads were processed to trim off primer/adaptor sequences and filtered by quality (QV > 25) using Trimmomatic (Bolger et al., 2014). Subsequently, all the clean reads were *de novo* assembled using the well-established short-read assembling Trinity pipeline (http://trinityrnaseq.github.io/) (Grabherr et al., 2011). Briefly, clean reads with a specified length of overlap were combined to produce longer contiguous sequences (contigs), and then these reads were mapped back onto the contigs with the paired-end information. The distance and relation among these contigs could be calculated based on paired-end reads, which allowed detection of contigs from the same transcript and also to calculate the distances among these contigs. Finally, the reconstructed contigs were further assembled and the constructed sequence that could not be extended on either end were considered as unique transcripts (UTs) (Wang et al., 2013).

### Functional annotation

The assembled UTs were annotated using BLASTX alignment against protein databases NCBI non-redundant (nr) (http://www.ncbi.nlm.nih.gov) and SwissProt (http://www.expasy.ch/sprot) (Magrane and Consortium, 2011), with an *e*-value cut-off < 10^−5^. For functional analysis of DEGs, the BLAST results based on hit score were imported into Blast2GO (Conesa et al., 2005) program to retrieve Gene Ontology (GO) annotation of DEGs for describing biological process, cellular component and molecular function categories. The distribution of DEGs functions was further classified with WEGO (Ye et al., 2006) software at the macro level. The DEGs sequences were also aligned to the Cluster of Orthologous Groups (COG) protein database (http://www.ncbi.nlm.nih.gov/COG/) to predict and classify possible functions. Furthermore, in order to elucidate the biological pathways represented in *P. minus* DEGs, the sequences obtained from Blast2GO were searched against Kyoto Encyclopedia of Genes and Genomes (KEGG) pathway maps database (Kanehisa and Goto, 2000).

### Differential gene expression analysis

RNA-seq by Expectation Maximization (RSEM) software package (Li and Dewey, 2011) was used to estimate transcript abundance. For each sample, the relative gene expression levels were normalized and expressed as FPKM (Fragments per Kilobase of Exon per Million reads Mapped) values. A Bioconductor package edgeR (Robinson et al., 2010) was used to analyze the differentially expressed genes by evaluating the dispersion of the entire dataset. UTs showing *P* ≤ 0.001, Benjamin-Hochberg False Discovery Rate (FDR) ≤ 0.05 and log2|fold change|> 2 were considered as significant differentially expressed genes (DEGs).

### Functional network analysis and KEGG pathway enrichment analysis

To assess major functional ontologies which are statistically overrepresented in the DEGs, global biological network analysis was performed using BiNGO (Biological Networks GO Ontology; v3.0.2), a Cytoscape plugin enrichment tool (Maere et al., 2005). GO-full terms within the BiNGO namespace with *P* ≤ 0.001 and Benjamini & Hochberg FDR correction were applied to depict the GO functional enrichment network and to show enriched functional categories in the up- and down-regulated DEGs.

To annotate putative pathways and biological functions involved in DEGs, the pathway enrichment analysis with hypergeometric test was carried out using “Annotate” and “Identify” programs in KEGG Orthology Based Annotation System (KOBAS 2.0) with Benjamini-Hochberg FDR correction (Xie et al., 2011). Up- and down-regulated DEGs were separately annotated against Arabidopsis database for enrichment analysis.

### Identification of transcription factors

To identify the transcription factors (TFs) in *P. minus*, the assembled reads were searched against known TFs, as grouped in Plant Transcription Factor Database (PlnTFDB), using default parameters and cut-off E-value of 1e^−5^. PlnTFDB is an integrative library of plant TFs which offers complete lists of TFs families that have fully sequenced genomes. Online protein sequences data for all species genes listed in the PlnTFDB database were downloaded from http://plntfdb.bio.uni-potsdam.de/v3.0/ website to align annotated UTs using local BLASTX. Moreover, a heatmap depicting the general trend of the differential expression of *P. minus* TF families in response to MeJA elicitation was created using MeV (http://mev.tm4.org/#/welcome) tool.

### qRT-PCR validation

The expression of 21 candidate genes was evaluated in relation to phenylpropanoid biosynthesis, JAs biosynthesis, JA signaling pathway and related TFs. Using PrimerBlast software, primers were designed to amplify short regions for each target and reference genes, ranging in size from 75 to 200 nt. Primers of 21 candidate UTs and reference gene are listed in Supplementary Table 1. For each sample, 1 μg DNase-treated (DNA-free™ DNase; Ambion, Huntingdon, UK) RNA was reverse transcribed using iScript™ cDNA Synthesis Kit (Bio-Rad, CA) according to the manufacturer's instruction. qRT-PCR was carried out by using iTaq Universal SYBR® Green SuperMix kit (Bio-Rad). The amplification was executed with the following cycling program: 3 min at 95°C, 40 cycles of 10 s at 95°C, 30 s at 60°C, and 30 s at 72°C; and 0.06 s at 65°C for reading. Real-time PCR was performed in the iQ™5 Real-Time PCR detection System (Bio-Rad, Hercules, CA). To find out the differences of relative fold for each sample, the CT value of each candidate genes was normalized to the CT values of reference gene CDPK (Calcium-Dependent Protein Kinase, Gene ID: comp58469_c0_seq3) and was calculated based upon the comparative CT (2^−ΔΔCt^) method (Schmittgen and Livak, 2008). The relative fold change for all candidate genes was set for both control and treated plants. Relative expression in RNA-seq was confirmed by three independent biological replicates and negative control of treated and mock-treated samples along with their three technical replicates.

## Results

### RNA sequencing, *De novo* assembly and annotation of *Polygonum minus* leaf transcriptome

We created a *P. minus* transcriptome dataset by sequencing RNA extracted from MeJA-treated and mock-treated leaf samples with two biological replicates each. The sequencing of four cDNA libraries on the Illumina HiSeq™ 2000 platform produced ~13.9 G bases of total nucleotides (192,167,972 raw short reads; Supplementary Table 2). After trimming the adaptors and low-quality reads, we obtained 191,565,800 high quality paired-end reads from four samples.

To study MeJA-regulated gene expression, the trimmed reads were *de novo* assembled using Trinity pipeline, generating 182,111 UTs with an average length of 537 bp, an N50 of 1387 bp and a GC percentage of 43.64% (Supplementary Table 3).

For annotation of assembled UTs, BLASTX annotation algorithm was used with an *E*-value threshold of 10^−5^. The sequence homology search which is based on sequence similarities to the public protein databases, was carried out against the nr and Swiss-Prot protein databases. Of all the UTs, 36.43% showed significant homology with nr database; and the E-value distributions of the top hits in the Swiss-Prot database indicated that 29.45% of the mapped sequences showed significant similarity.

### Differentially expressed genes in response to MeJA

The abundance of UTs was estimated by mapping back the trimmed reads against the UTs with RSEM and their expression levels were represented as FPKM values. The two biological replicates of both control and MeJA-treated samples showed high correlation (*R*^2^ > 0.79) in FPKM values (Supplementary Figure S1). Among 182,111 assembled UTs, a total of 2374 DEGs were found to be significantly differentially expressed at *P* ≤ 0.001, FDR ≤ 0.05, and log2|fold change|> 2 in response to MeJA treatment, of which 1419 were up-regulated and 955 were down-regulated. This suggests that the MeJA-elicited response involves more gene activation than suppression.

### Functional annotation and gene ontology classification of DEGs

**Figure 1:**
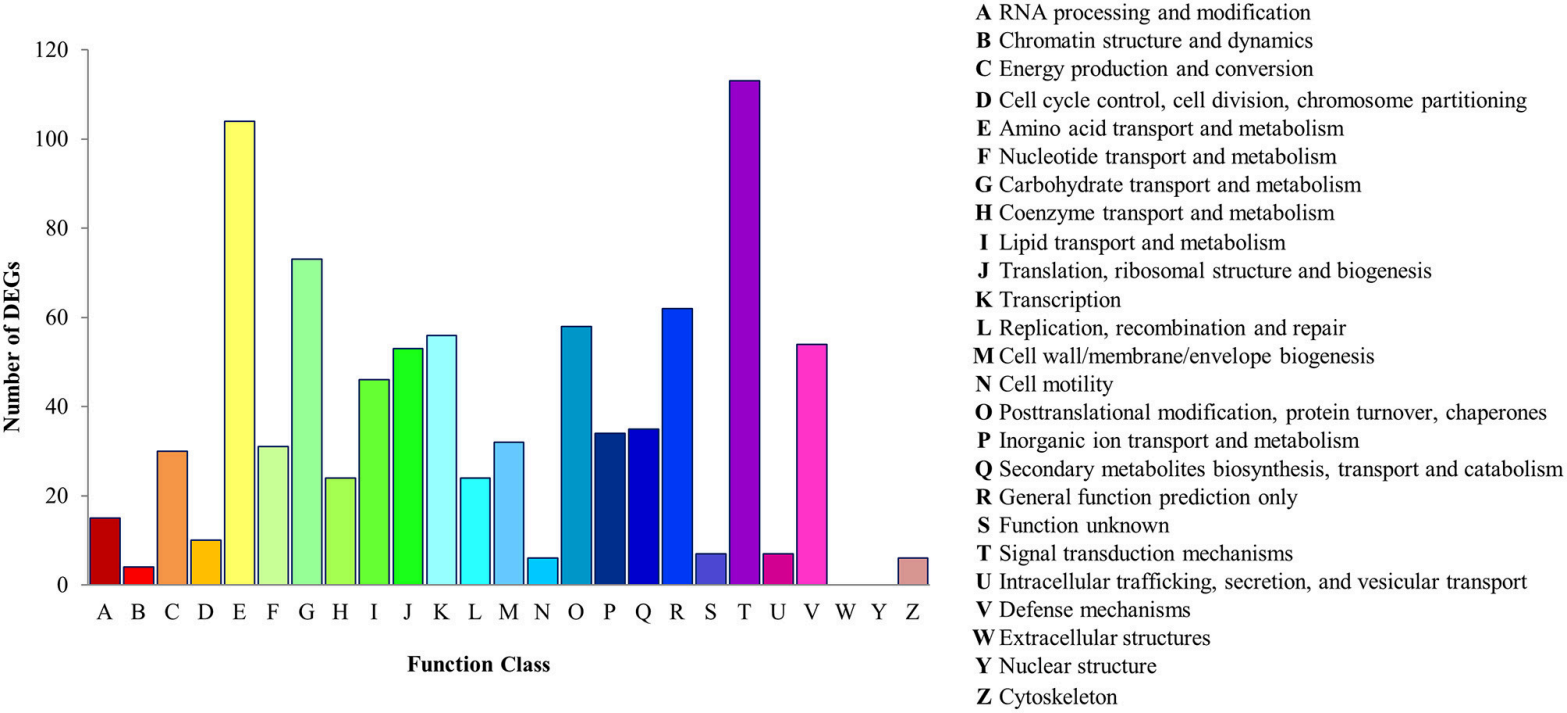
**Histogram presentation of COG functional distribution of the ***P. minus*** leaf DEGs**.

For the prediction and classification of possible functions, all DEGs were aligned to the COG database (Tatusov et al., 2003). A total of 884 DEGs were classified into 23 COG functional categories, among which the category “Signal transduction mechanisms” (113, 12.78%) was predominant cluster that suggesting the differentially regulation of signal transduction pathways, particularly the transduction of plant hormones. In addition, a high percentage of genes were also assigned to “Amino acid transport and metabolism” (104, 11.76%) followed by “Carbohydrate transport and metabolism” (73, 8.26%) and “General function prediction only” (62, 7.01%). Only a few genes were assigned to the categories “Intracellular trafficking, secretion, and vesicular transport,” “Cytoskeleton,” “Cell motility,” and “Chromatin structure and dynamics.” Analysis of COG classification indicated that the identified genes are involved in different biological processes. For example, 54 and 35 genes were respectively annotated to “Defense mechanisms” and “Secondary metabolites biosynthesis, transport and catabolism,” and are therefore associated with defense and biosynthesis of secondary metabolism pathways. Hence, the identification of genes in these pathways is required for the elucidation of the molecular mechanisms orchestrating stress response induction. The results of COG functional annotation of the *P. minus* DEGs are depicted in Figure 1. Further, DEGs with nr annotation was also annotated and classified under GO analysis to classify the functions of the predicted differential expressed genes in *P. minus*.

**Figure 2:**
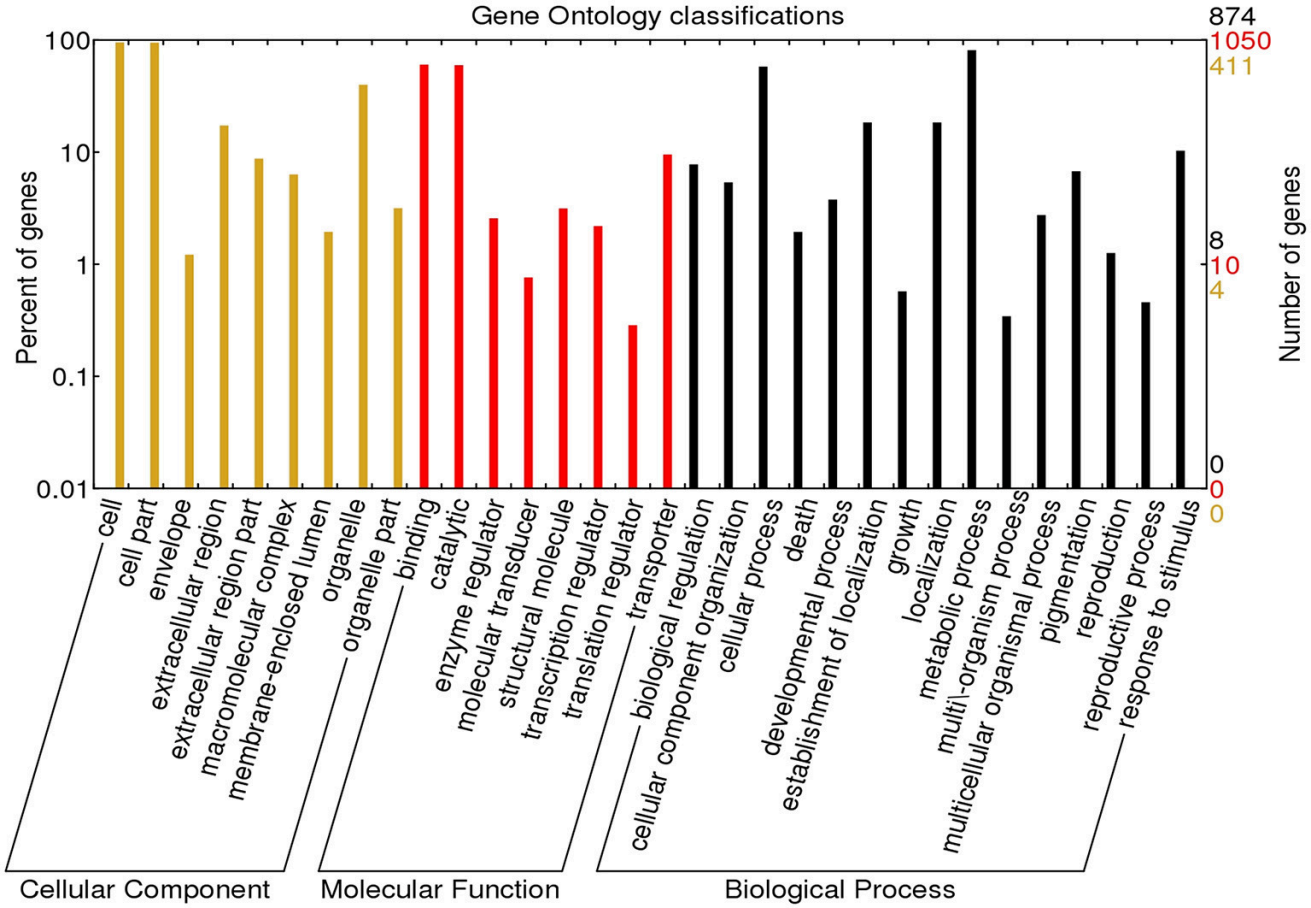
**Gene Ontology functional classification of ***P. minus*** leaf transcriptome**. The results are summarized in three GO categories: Biological process, molecular function, and cellular component. The right y-axis indicates the number of DEGs in each category and the left y-axis indicates the percentage of sequences in the same category.

Using Blast2GO (Conesa et al., 2005), a total of 1178 DEGs were assigned with 205 GO terms. The majority of the top hits belonged to protein sequences of *Beta vulgaris*, followed by *Vitis vinifera, Theobroma cacao, Nelumbo nucifera*, and *Jatropha curcas* (Supplementary Figure S2). As shown in Figure 2, the assignments to biological processes (109, 53.17%) were mostly metabolic and cellular processes (710 and 506 DEGs respectively, Supplementary File 1; Datasheet 1). Notably, 116 DEGs were assigned to responses to stimulus. The highest percentages of GO terms under cellular component category (52, 25.37%) were in cell and cell part (250 and 247 DEGs respectively, Supplementary File 1; Datasheet 2). In the molecular function category (44, 21.46%), the majority of the GO terms were grouped into binding and catalytic activity (634 and 627 DEGs respectively, Supplementary File 1; Datasheet 3). GO classification of up- and down-regulated DEGs were shown in Supplementary Figure S3.

### Go-based functional network analysis of DEGs

The derived graphs from BINGO represent specific functions which were significantly enriched in three gene ontology categories. From the enrichment analysis, we identified biologically informative GO terms for comparison between the up- and down-regulated DEGs. The representative graph for the up-regulated DEGs (Supplementary Figure S4A) indicated that most of the GO terms were significantly overrepresented in the defense and protective functions for plant response to chemical stimulus, stress and wounding. Secondary metabolic processes such as biosynthesis of phenylpropanoid, flavonoid, amino acid derivative, and aromatic compound were overrepresented. Concomitantly, under the molecular function category, DEGs related to “transferase,” “transporter,” “oxidoreductase,” “lyase,” and “transcription repressor” activities were significantly up-regulated 24 h after MeJA elicitation.

In contrast, majority GO terms of the down-regulated DEGs were significantly enriched in biological process related to photosynthesis, response to abiotic and biotic stimuli, respectively to light and bacterium, and generation of precursor metabolites and energy (Supplementary Figure S4B). In the molecular function category, GO terms related to “catalytic activity” of oxidoreductase and transferase, and “binding” of chlorophyll were represented in down-regulated DEGs.

### KEGG pathway-based gene classification

According to sequence homology searches using BLASTX against the KEGG pathway database, 366 of 2374 DEGs were assigned to 3 main categories of 82 KEGG pathways representing compound biosynthesis, metabolism and degradation. In addition, 415 sequences of *P. minus* leaf were mapped to 193 enzymes (Supplementary Table 4). As shown in Table 1, the category with the greatest number of mapped entry was metabolism with the most in carbohydrate metabolism (89), followed by amino acid metabolism (49), energy metabolism (40), biosynthesis of other secondary metabolites (29), and lipid metabolism (27). Notably, the most represented pathways in secondary metabolism were those of phenylpropanoid and flavonoid biosynthesis (Supplementary Table 4). Only a few DEGs were mapped to translation (2) and signal transduction (1) categories.

**Table 1:** **Distributions of differentially expressed genes (DEGs) in KEGG pathway database classification**.

**Category**	**Sub-category**	**DEGs with pathway annotation**
Metabolism	Global and overview maps	49
	Carbohydrate metabolism	89
	Energy metabolism	40
	Lipid metabolism	27
	Nucleotide metabolism	27
	Amino acid metabolism	49
	Metabolism of other amino acids	8
	Glycan biosynthesis and metabolism	9
	Metabolism of cofactors and vitamins	19
	Metabolism of terpenoids and polyketides	4
	Biosynthesis of other secondary metabolites	29
	Xenobiotics biodegradation and metabolism	13
Genetic information processing	Translation	2
Environmental information processing	Signal transduction	1
Total unique transcripts		366

### KEGG pathway enrichment analysis

KEGG pathway enrichment analysis was conducted using KOBAS to identify statistically significantly enriched pathways in response to MeJA elicitation. We identified 289 up-regulated and 180 down-regulated DEGs which were significantly enriched in eight and nine KEGG pathways respectively. The most significant with a majority number of up regulated DEGs involved in protective activities pathways such as biosynthesis of secondary metabolites, phenylpropanoid biosynthesis, and phenylalanine metabolism (Table 2). This is consistent with the finding from KEGG pathway mapping that greater number of up-regulated DEGs were found in phenylalanine, tyrosine and tryptophan biosynthesis (15), phenylalanine metabolism (13), phenylpropanoid biosynthesis (13), flavonoid biosynthesis (8), and α-linolenic acid metabolism (4) (Supplementary Table 5).

**Table 2:** **KEGG pathway enrichment of up- and down-regulated transcripts in MeJA-elicited ***P. minus*** leaf**.

**KEGG pathway**	**Input number**	**Background number**	***P*****-Value**	**Corrected *P*-Value**
**UP-REGULATED DEGS**				
KO01110-Biosynthesis of secondary metabolites	153	995	1.84E-08	9.60E-07
KO00940-Phenylpropanoid biosynthesis	40	154	1.81E-07	7.69E-06
KO00360-Phenylalanine metabolism	31	114	1.93E-06	6.68E-05
KO00400-Phenylalanine, tyrosine, and tryptophan biosynthesis	20	57	6.05E-06	0.000177
KO00592-alpha-Linolenic acid metabolism	15	33	7.69E-06	0.000218
KO00280-Valine, leucine, and isoleucine degradation	12	45	3.12E-03	0.029688
KO00071-Fatty acid degradation	11	40	3.76E-03	0.034722
KO00966-Glucosinolate biosynthesis	7	19	5.52E-03	0.046785
**DOWN-REGULATED DEGS**				
KO00195-Photosynthesis	34	77	1.95E-17	1.07E-15
KO00196-Photosynthesis—antenna proteins	17	22	3.25E-12	1.18E-10
KO00630-Glyoxylate and dicarboxylate metabolism	22	63	3.78E-10	9.90E-09
KO00710-Carbon fixation in photosynthetic organisms	20	69	3.29E-08	6.53E-07
KO01200-Carbon metabolism	35	243	1.95E-06	3.27E-05
KO00260-Glycine, serine, and threonine metabolism	16	69	9.20E-06	0.000142
KO00910-Nitrogen metabolism	12	42	2.13E-05	0.000307
KO00250-Alanine, aspartate, and glutamate metabolism	12	48	6.47E-05	0.000859
KO04146-Peroxisome	12	81	0.003866	0.033538

Conversely, photosynthesis, photosynthesis-antenna proteins as well as glyoxylate and dicarboxylate metabolism were amongst the significantly enriched pathways for down-regulated DEGs (Table 2). In the KEGG pathway mapping, more down-regulated DEGs were also mapped to carbon fixation in photosynthetic organisms (13), fructose, and mannose metabolism (9), glycolysis/gluconeogenesis (9), pentose phosphate pathway (9), starch and sucrose metabolism (8), glyoxylate and dicarboxylate metabolism (7), and porphyrin and chlorophyll metabolism (4) (Supplementary Table 5). Such growth-defense trade-offs presumably contribute to improvement in plant fitness through the diversion of saved energy from down-regulated photosynthesis to pertinent defense responses in changing environments (Rojas et al., 2014).

### Identification of differentially expressed MeJA-responsive transcription factors

**Figure 3:**
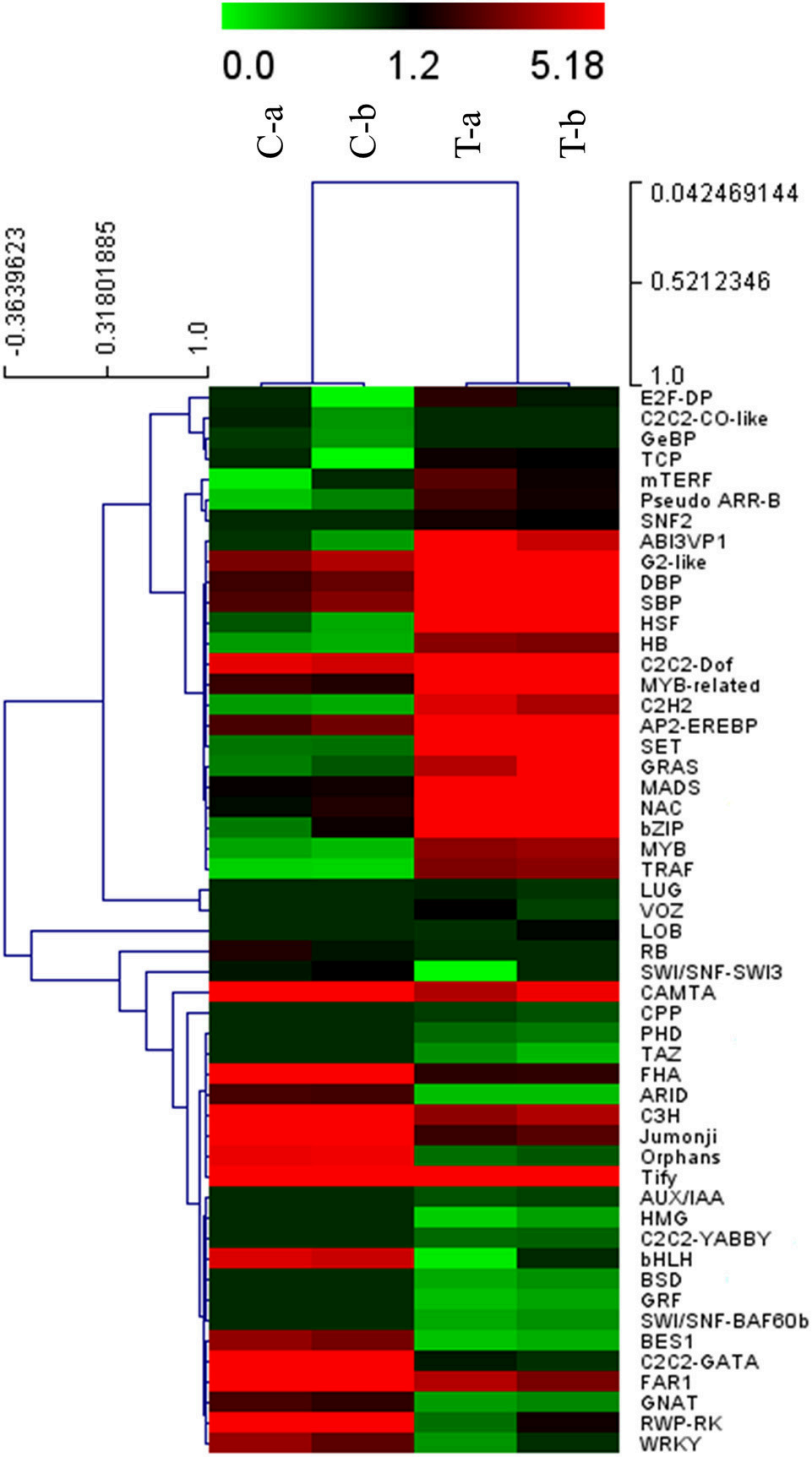
**Hierarchical cluster analysis of differentially expressed putative TFs in ***P. minus*** leaf in response to MeJA**. Several transcription factor families indicating differential expression are shown on the right side. The x-axis represents each replicate of control and MeJA treated conditions. The y-axis refers to TF genes expression levels. C, conrol; T, treated.

A total of 2374 DEGs were searched against the Plant Transcription Factor Database (PlnTFDB) and 472 transcripts upon MeJA elicitation were significantly annotated as differentially expressed TFs. These TFs were grouped into 54 families including one orphan family containing 14 TFs which could not be grouped in any TF family. Most of the differentially expressed TFs are involved in plant stress responses, secondary metabolism and development processes, such as up-regulated expression of AP2/ERF, MYB, MADS, and NAC families, while bHLH and WRKY families were down-regulated (Figure 3). Members of these TF families have been studied and noted as candidate genes regulating the stress responses in different species (Van Verk et al., 2009; Yang et al., 2012a). The top 20 annotated differentially expressed TF families of *P. minus* leaf in response to MeJA treatment are shown in Supplementary Figure S5.

In addition, our results indicated that among the up-regulated TF families (Supplementary Table 6), the MYB family contained the largest number of DEGs (19 members). MYB TFs represent one of the largest plant families that have important functions in various physiological processes under different conditions. These TFs are involved in primary and secondary metabolism, hormone signal transduction, defense response to environmental stresses and development process, etc. In recent years, several MYB genes were shown to be involved in the regulation of phenylpropanoid metabolism (Md-Mustafa et al., 2014; Liu et al., 2015). The Arabidopsis AtMB12 protein was identified as a specific regulator of phenylpropanoid biosynthesis of flavonol (Mehrtens et al., 2005). In tobacco MeJA signal transduction, NtMYBJS1 protein was shown to regulate some of early phenylpropanoid-related genes leading to the enhanced accumulation of phenylpropanoid conjugates under stress (Gális et al., 2006), and these results are consistent with our RNA-seq data.

The most abundant down-regulated TF (Supplementary Table 6) belonged to FAR1 (FAR-RED IMPAIRED RESPONSE1) family. This family is regarded as plant-specific positive regulators of phytochrome A signal transduction pathway that able to absorb far-red (FR) light (Hudson et al., 1999). Interestingly, FR light related-responses genes were significantly down-regulated in MeJA-elicited *P. minus* leaf (Supplementary Figure S4).

Differentially expressed TFs which respond to MeJA treatment demonstrated that transcription regulation played a main role in MeJA-induced response network. Moreover, these results support previous studies which revealed the interaction of stress responses and developmental process (Chen et al., 2002; Cooper et al., 2003).

### Validation of RNA-Seq analysis by qRT-PCR

**Figure 4:**
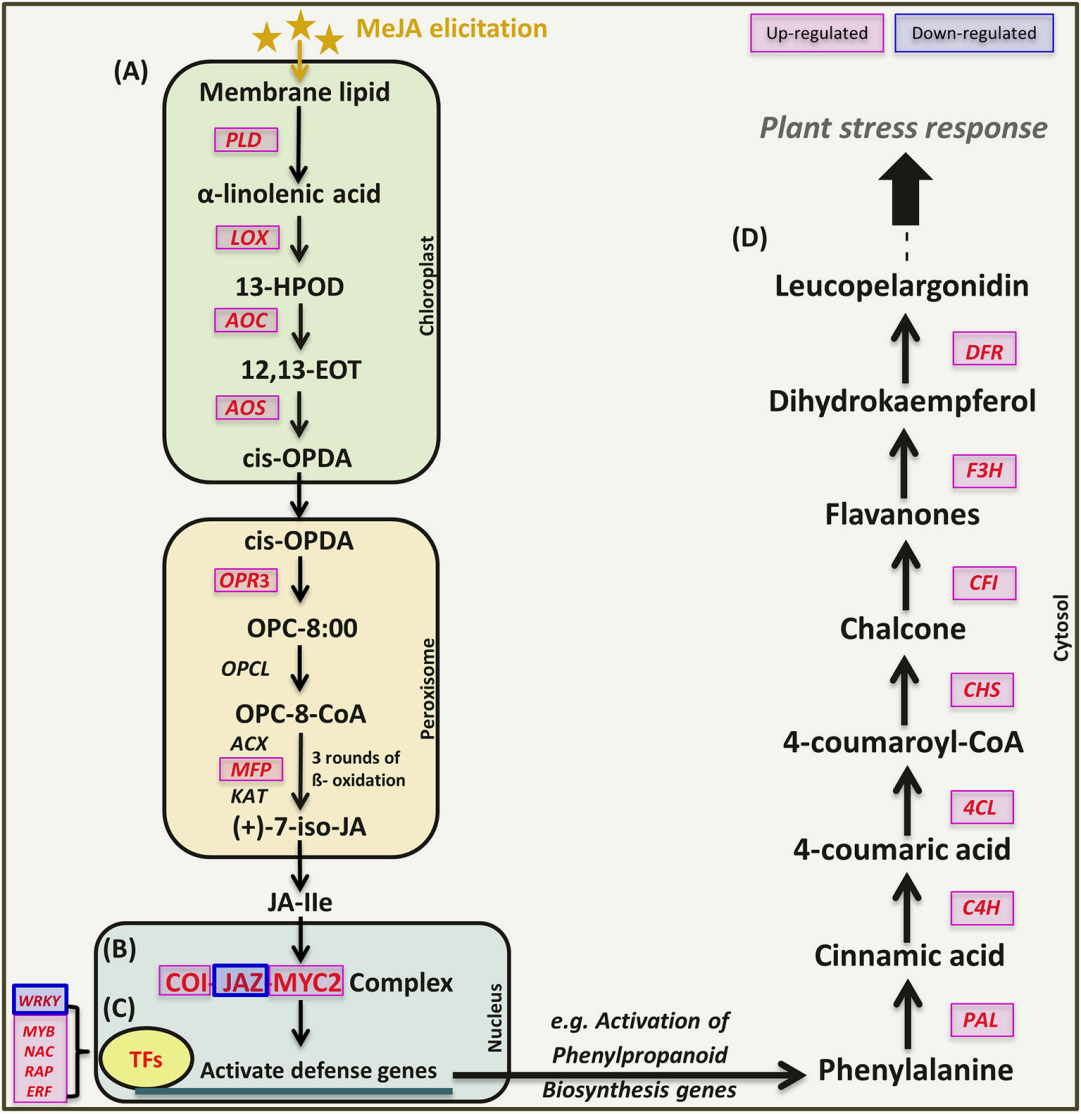
**Schematic representation of jasmonic acid signaling and activation of defense genes (phenylpropanoids biosynthesis) in response to MeJA elicitation**. Genes marked in red were validated by qRT-PCR in this study. Figure modified from (Ge et al., 2015). **(A)** JA biosynthesis, **(B)** JA signaling pathway, **(C)** Transcription factors, and **(D)** Phenylpropanoids pathway.

**Figure 5:**
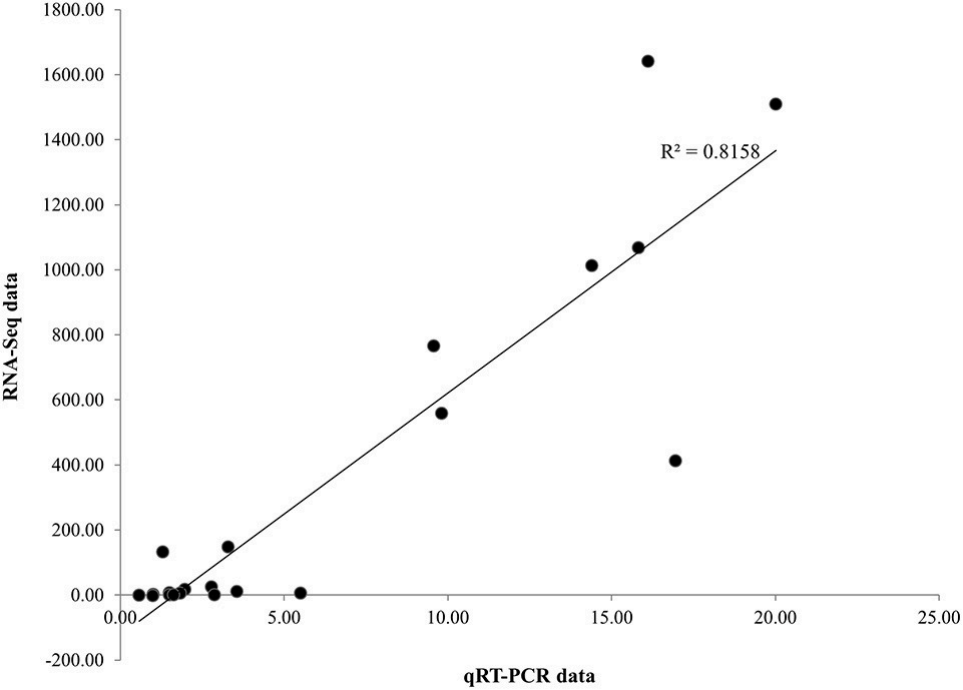
**Scatter plot of fold change coefficient analysis**. Illustrating the positive correlation of fold change of 21 selected genes expression ratio between qRT-PCR and RNA-seq data.

To evaluate our transcriptome result, we performed qRT-PCR to confirm the expression levels of 21 DEGs involved in the biosynthesis of phenylpropanoids and jasmonates, as well as JA signaling pathways and related TFs (Figure 4). qRT-PCR analysis revealed considerable activation for most of the candidate genes (except WRKY and NAC) in *P. minus* leaves upon elicitation which is consistent and correlates well (*R*^2^ > 0.82) with the RNA-seq data (Figure 5).

## Discussion

Since plants produce various volatile secondary metabolites to cope with changing environments against oxidative and other stresses, plant chemical elicitors such as MeJA was applied to induce a defense response. Understanding the molecular mechanism underlying MeJA that triggers plant defense response is key to enhancing secondary metabolite production. *P. minus* remains understudied despite being one of the most commonly used aromatic and medicinal plants in Southeast Asia.

In a previous study, using a cDNA-AFLP approach, Ee et al. (2013) identified significant changes in gene expression of 52 transcript-derived fragments (TDFs) of *P. minus* leaf resulting from MeJA treatment, including those involved in stress response (6) and secondary metabolic processes (1). However, cDNA-AFLP study is hindered by the limited number of sequenced fragments. RNA-seq approach allows global and more accurate quantification of gene expression to obtain a better picture of gene regulation with more identified DEGs.

Leaf samples harvested 24 h after MeJA-elicitation were chosen for RNA-seq analysis based on results from metabolite profiling, which showed the greatest response (data not shown). We obtained 192 million clean reads which were assembled *de novo* into 182,111 UTs. The number of assembled UTs in *P. minus* is comparable to *de novo* transcriptome assembly of cotton, perennial ryegrass and *Camellia sinensis* using the same sequencing platform and assembler (Xu et al., 2013; Farrell et al., 2014; Wu et al., 2014). The similarity searches of UTs against public protein databases found that majority (63.57%) UTs without functional annotation due to the lack of matching homologous sequences. These UTs with unknown function could be candidates of novel genes for future studies.

DEG analysis found 2374 UTs as statistically significant DEGs in MeJA-elicited *P. minus* leaf. DEGs were assigned to a broad range of COG classifications and GO categories suggesting that DEGs represent a variety of transcripts in *P. minus* leaf transcriptome. Among the 23 functional categories identified, we found the category “signal transduction mechanisms” to be the most represented in COG classification accounting for its need to induce the expression of specific subset of defense genes that result in the activation of the overall defense reaction (Pérez-Clemente et al., 2012). A large number of DEGs were annotated to the categories “Amino acid transport and metabolism,” “Carbohydrate transport and metabolism,” and “General function prediction only.” Pathways involving in transport and metabolism of amino acid and carbohydrate are involved in plant growth and response to various stresses (Gupta and Kaur, 2005; Less and Galili, 2008). In addition amino acids are tightly linked to carbohydrate metabolism and serve as precursors for the synthesis of various classes of secondary metabolites involved in defense mechanism (Pratelli and Pilot, 2014).

GO-based functional network analysis of DEGs showed GO terms related to “response to stress” involving the biosynthesis of primary and secondary metabolites were enriched in the up-regulated DEGs, whereas terms related to “cell structure” and “growth” such as photosynthesis, response to radiation, light reaction and photosynthetic electron transport chain were enriched in the down-regulated DEGs (Supplementary Figure S4). Light provides energy needed for photosynthesis and plays a role in photoregulation of plant growth and development (Diffey, 2013). These results support the idea that down-regulation of photosynthetic genes and their corresponding proteins as well as reduction in electron transport chain is a conserved feature of plant responses to many environmental stresses, however, the physiological significance of this phenomenon remains unclear and no experimental evidence is available to explain why it happens (Attaran et al., 2014; Rojas et al., 2014).

The KEGG database was searched to identify the biological pathways operating in MeJA-elicited *P. minus* leaves. Genes in the KEGG metabolism categories including starch and sucrose metabolism and pentose phosphate pathways with important functions in plant growth and development, as well as flavonoid and phenylpropanoid biosynthetic pathways important for stress responses were enriched. These secondary metabolites were previously reported to accumulate in high levels in sweet basil after MeJA treatment (Ku and Juvik, 2013; Misra et al., 2014).

The pathway enrichment analysis results of differentially MeJA-expressed genes can give helpful information to study specific functions, mechanisms and pathways that have important roles in MeJA response in *P. minus* leaves. Pathway-based analysis highlights that genes within similar pathways commonly cooperate to operate certain biological functions (Wenping et al., 2011). This allows us to gain insight into the functions of these genes in the leaf transcriptome of *P. minus*. We found that genes related to the defense response were up- regulated, while the genes associated with growth and development processes were down-regulated upon MeJA elicitation. (Supplementary Figure S3). These collective findings from different functional annotation (KEGG, GO, and COG) of DEGs also support the view that the triggering defense responses by allocating resources from growth to defense lead to the negative effect on photosynthesis, and consistent with findings that plant stress hormone JAs plays a central role in controlling resource allocation between the competing processes of defense and growth (Attaran et al., 2014).

Most of the genes associated with lipid metabolism, such as alpha-linolenic acid metabolism, biosynthesis of unsaturated fatty acids, fatty acid biosynthesis/degradation, and linoleic acid metabolism displayed differential expression in response to MeJA. Four DEGs were found to be associated with the alpha-linolenic acid metabolism (Supplementary Table 4) that results in JA biosynthesis, including oxidase (EC: 1.3.3.6) and 13S-lipoxygenase (EC: 1.13.11.12) (Supplementary Figure S6). The unsaturated fatty acid alpha-linolenic acid is conserved in jasmonates production which is involved in transcriptional regulation of defense genes in response to environmental changes (Conconi et al., 1996). This result suggests the presence of a regulatory positive feedback mechanism for the JA biosynthesis (Sasaki et al., 2001).

Exogenous MeJA was reported by many studies to induce the expression of genes encoding JA biosynthetic enzymes and the JA signaling pathway in some plant species such as tomato, Arabidopsis and tobacco (Maucher et al., 2000; Chen et al., 2006; Kazan and Manners, 2008). In JA signaling pathway, the F-box protein CORONATINE INSENSITIVE 1 (COI1) bind to members of the JA ZIM domain (JAZ) repressor protein in the presence of JA-isoleucine (JA-Ile), and marks the complex for degradation by the 26S proteasome which releases the MYC2 TFs to activate transcription of JA-inducible genes (Wasternack and Hause, 2013).

**Figure 6:**
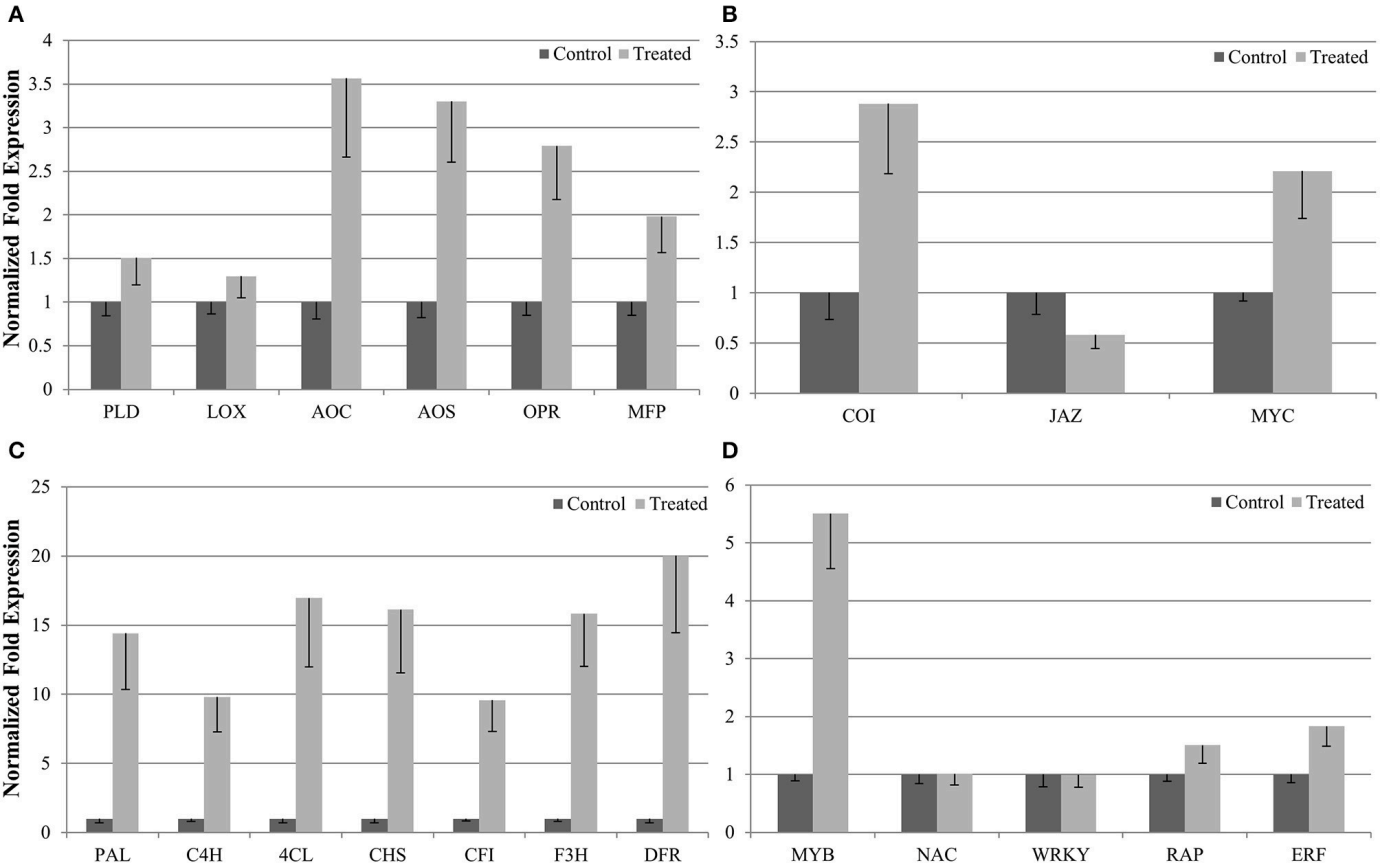
**qRT-PCR validation of differentially expressed genes**. Expression of 21 selected UTs involved in **(A)** JA biosynthesis, **(B)** JA signaling pathway, **(C)** phenylpropanoids pathway, and **(D)** transcription factors. were validated using qRT-PCR and compared with their expression obtained from RNA-seq. Values were normalized relative to the expression level of reference gene in the same cDNA sample. Data are the means (± SE) of three technical replicates of three biological replicates.

The expression of six genes (LOX, AOS, AOC, PLD, OPR, and MFP) in JA biosynthesis (Figure 6A) and three related genes (COI1, JAZ, MYC2) in the JA signaling pathway (Figure 6B) were consistent with the mechanisms observed in other plant species (Turner et al., 2002; Chini et al., 2009). This indicates that exogenous MeJA modulates JA biosynthesis and JA signaling pathway, which further regulate downstream gene expression. Genes related to key enzymes in phenylpropanoid pathway (Figure 6C) upstream of the flavonoid branch such as L-phenylalanine ammonia lyase (PAL), cinnamate 4-hydoxylase (C4H), 4-coumarate CoA ligase (4CL), chalcone synthase (CHS), chalcone flavanone isomerase (CFI), flavanone 3-hydroxylase (F3H), and dihydroflavonol reductase (DFR) as well as number of genes encoding TFs were all induced by MeJA elicitation (Figure 6D).

Phytohormones such as JAs play key roles in plants responding to environmental cues to regulate plant growth and development under different stresses (Miao et al., 2015). Response of cellular functions to environmental cues requires differential gene expression, which is regulated by TFs. In plant cells, external signals modulate the levels of these regulatory elements which in turn regulate the transcription of defense genes in response to stress (Vom Endt et al., 2002), e.g., the NAC TFs have been shown to regulates JA-induced expression of defense genes in *Arabidopsis thaliana* (Bu et al., 2008). It has been suggested that stress-responsive TFs might be involved in stress tolerance (Saibo et al., 2009).

Since TFs play significant roles in stress signaling and plant defensive responses by translating signals from stresses into changes in gene expression (Lindemose et al., 2013), we executed comprehensive analysis of the differentially expressed TFs that regulate biosynthesis of MeJA-responsive genes. Based on the annotated UTs, 2.27% of the DEGs in the *P. minus* leaf encode TFs. As JA is essential in mediating plants defense responses and the induction of secondary metabolite synthesis (Shoji and Hashimoto, 2013), we hypothesized that those TFs involved in the regulation of defensive secondary metabolite biosynthesis can also induce JA genes. Interestingly, majority of *P. minus* TF families induced by MeJA elicitation mainly associated with either a specific hormones (e.g., jasmonic acid and salicylic acid) pathway such as AP2-EREBP family or a general stress response of MYB, NAC, MYC, bHLH, and WRKY families. These TF families were previously shown as jasmonate responsive transcriptional regulators of plant defensive gene biosynthesis (Chatel et al., 2003; Gális et al., 2006; Bu et al., 2008; Yu et al., 2012; Schluttenhofer et al., 2014), and suggesting their possible roles in the regulation of secondary metabolite biosynthesis in *P. minus* leaf. The largest class of TFs down regulated by MeJA elicitation was the FAR1 TF family. Photosynthesis and related metabolism are affected under environmental stresses (Saibo et al., 2009) and FAR1 family is known as a regulator of chlorophyll biosynthesis that serve as major pigments in photosynthesis and chloroplast division which is the exclusive location of photosynthesis (Tang et al., 2013; Wang and Wang, 2015). Thus, the result has been proposed that FAR1 family may have a role in down-regulating photosynthetic genes in *P. minus* leaf under MeJA treatment. Additionally, in our data, the largest class of TFs up-regulated by MeJA stress was the MYB TF family, suggesting that this TF family plays a major role in MeJA stress tolerance besides its role in the regulation of genes related to phenylpropanoid biosynthesis.

Among the plant secondary metabolites, phenylpropanoids are a group of aromatic amino acid phenylalanine derived physiologically active secondary metabolites such as, anthocyanins and isoflavonoids (Zhao et al., 2013). Phenylpropanoids contribute to the scents/aromas and play an important role in all aspects of plant responses against biotic and abiotic stimuli (Van Moerkercke et al., 2012). In our DEGs study, treatment by MeJA affected the pathways contributing to phenylpropanoid biosynthesis, phenylalanine metabolism, and also phenylalanine, tyrosine, and tryptophan biosynthesis.

In the pathway of phenylpropanoid biosynthesis, the expression of phenylalanine ammonia-lyase (EC:4.3.1.24) induced by 3.15-fold, which might lead directly to more cinnamic acid synthesis. Additionally, the high expression of gentiobiase (betaglucosidase, EC: 3.2.1.21) and lactoperoxidase (EC:1.11.1.7) in this pathway could result in enhanced content of coumarin and lignin-derived components respectively (Supplementary Figure S7), which were previously shown to be MeJA-induced (Ramakrishna and Ravishankar, 2011; Mandal, 2015). Furthermore, flavonoid components are the most common plant pigments which contribute to the plant stress responses (Falcone Ferreyra et al., 2012) and MeJA also affects the biosynthesis of flavonoid-derived volatiles. The transcriptome results showed that several DEGs involved in the flavonoid pathway were affected by MeJA elicitation. The expressions of chalcone isomerase (EC: 5.5.1.6), flavanone synthase (EC: 2.3.1.74), and 4-reductase (EC: 1.1.1.219) increased in MeJA-treated *P. minus* leaves. Increased expression of chalcone isomerase and flavanone synthase might result in up-regulated biosynthesis of pinocembrin as well as naringenin, and the leucopelargonidin content could be affected by 4-reductase-induced expression (Supplementary Figure S8).

## Conclusion

In this study of MeJA-elicited *P. minus* leaf transcriptome, 182,111 UTs were assembled with 2374 DEGs identified. The is the first RNA-seq study on the response to MeJA of a non-model plant from Polygonaceae family, which comprises many useful medicinal plants. Functional annotation of DEGs based on COG, GO and KEGG showed the upregulation of UTs involve in plant defense and protective functions, as well as secondary metabolic processes. Conversely, UTs related to photosynthesis were down-regulated. This is in accordance to the TF families found to be differentially expressed. MeJA elicitation is found to upregulate endogenous JA biosynthesis. The molecular mechanism on how MeJA triggers the upregulation of phenylpropanoid pathway is proposed and validated by qRT-PCR analysis. These results support the physiological trade-offs between stress response and growth despite that the underlying mechanism remains unclear. Furthermore, current *P. minus* transcriptome provides an extensive sequence resource for gene discovery and functional studies in related species.

## Author contributions

NN and HG conceived the project. NN supervised the research work and contributed reagents/materials/analysis tools. HG designed the experiments, supervised the analysis, and critically revised the manuscript. KL conducted bioinformatics for raw RNA-seq data. RR collected plant materials, performed stress treatment, prepared samples for RNA-seq, conducted the gene functional analysis and TF analysis, performed qPCR based expression analysis and wrote the manuscript.

### Conflict of interest statement

The authors declare that the research was conducted in the absence of any commercial or financial relationships that could be construed as a potential conflict of interest.

## References

[B1] Aranega-Bou P. de la O. L. M. Finiti I. García-Agustín P. González-Bosch C. (2014). Priming of plant resistance by natural compounds. Hexanoic acid as a model. Front. Plant Sci. 5:448. 10.3389/fpls.2014.00488 25324848 PMC4181288

[B2] Attaran E. Major I. T. Cruz J. A. Rosa B. A. Koo A. J. Chen J.. (2014). Temporal dynamics of growth and photosynthesis suppression in response to jasmonate signaling. Plant Physiol. 165, 1302–1314. 10.1104/pp.114.239004 24820026 PMC4081338

[B3] Baharum S. N. Bunawan H. Ghani M. A. Mustapha W. A. W. Noor N. M. (2010). Analysis of the chemical composition of the essential oil of *Polygonum minus* Huds. using two-dimensional gas chromatography-time-of-flight mass spectrometry (GC-TOF MS). Molecules 15, 7006–7015. 10.3390/molecules15107006 20944520 PMC6259174

[B4] Bolger A. M. Lohse M. Usadel B. (2014). Trimmomatic: a flexible trimmer for Illumina sequence data. Bioinformatics btu170. 30, 2114–2120. 10.1093/bioinformatics/btu170 24695404 PMC4103590

[B5] Bu Q. Jiang H. Li C.-B. Zhai Q. Zhang J. Wu X.. (2008). Role of the *Arabidopsis thaliana* NAC transcription factors ANAC019 and ANAC055 in regulating jasmonic acid-signaled defense responses. Cell Res. 18, 756–767. 10.1038/cr.2008.53 18427573

[B6] Chatel G. Montiel G. Pré M. Memelink J. Thiersault M. Saint-Pierre B.. (2003). CrMYC1, a Catharanthus roseus elicitor-and jasmonate-responsive bHLH transcription factor that binds the G-box element of the strictosidine synthase gene promoter*. J. Exp. Bot. 54, 2587–2588. 10.1093/jxb/erg275 12966042

[B7] Chen H. Jones A. D. Howe G. A. (2006). Constitutive activation of the jasmonate signaling pathway enhances the production of secondary metabolites in tomato. FEBS Lett. 580, 2540–2546. 10.1016/j.febslet.2006.03.070 16647069

[B8] Chen W. Provart N. J. Glazebrook J. Katagiri F. Chang H.-S. Eulgem T.. (2002). Expression profile matrix of Arabidopsis transcription factor genes suggests their putative functions in response to environmental stresses. Plant Cell 14, 559–574. 10.1105/tpc.010410 11910004 PMC150579

[B9] Chini A. Boter M. Solano R. (2009). Plant oxylipins: COI1/JAZs/MYC2 as the core jasmonic acid-signalling module. FEBS J. 276, 4682–4692. 10.1111/j.1742-4658.2009.07194.x 19663905

[B10] Christapher P. V. Parasuraman S. Christina J. M. A. Asmawi M. Z. Vikneswaran M. (2015). Review on *Polygonum minus*. Huds, a commonly used food additive in Southeast Asia. Pharmacogn. Res. 7, 1. 10.4103/0974-8490.147125 25598627 PMC4285636

[B11] Cocetta G. Rossoni M. Gardana C. Mignani I. Ferrante A. Spinardi A. (2015). Methyl jasmonate affects phenolic metabolism and gene expression in blueberry (*Vaccinium corymbosum*). Physiol. Plant 153, 269–283. 10.1111/ppl.12243 24943920

[B12] Conconi A. Miquel M. Ryan C. (1996). Intracellular levels of free linolenic and linoleic acids increase in tomato leaves in response to wounding. Plant Physiol. 111, 797–803. 10.1104/pp.111.3.797 12226331 PMC157897

[B13] Conesa A. Götz S. García-Gómez J. M. Terol J. Talón M. Robles M. (2005). Blast2GO: a universal tool for annotation, visualization and analysis in functional genomics research. Bioinformatics 21, 3674–3676. 10.1093/bioinformatics/bti610 16081474

[B14] Cooper B. Clarke J. D. Budworth P. Kreps J. Hutchison D. Park S.. (2003). A network of rice genes associated with stress response and seed development. Proc. Natl. Acad. Sci. U.S.A. 100, 4945–4950. 10.1073/pnas.0737574100 12684538 PMC153660

[B15] Crozier A. Clifford M. N. Ashihara H. (2008). Plant Secondary Metabolites: Occurrence, Structure and Role in the Human Diet. Oxford: John Wiley & Sons.

[B16] Davies K. M. Schwinn K. E. (2003). Transcriptional regulation of secondary metabolism. Funct. Plant Biol. 30, 913–925. 10.1071/FP03062 32689076

[B17] De Geyter N. Gholami A. Goormachtig S. Goossens A. (2012). Transcriptional machineries in jasmonate-elicited plant secondary metabolism. Trends Plant Sci. 17, 349–359. 10.1016/j.tplants.2012.03.001 22459758

[B18] Diffey B. L. (2013). Radiation Measurement in Photobiology. San Diego, CA: Academic press.

[B19] Dudareva N. Klempien A. Muhlemann J. K. Kaplan I. (2013). Biosynthesis, function and metabolic engineering of plant volatile organic compounds. New Phytol. 198, 16–32. 10.1111/nph.12145 23383981

[B20] Ee S.-F. Oh J.-M. Noor N. M. Kwon T.-R. Mohamed-Hussein Z.-A. Ismail I.. (2013). Transcriptome profiling of genes induced by salicylic acid and methyl jasmonate in *Polygonum minus*. Mol. Biol. Rep. 40, 2231–2241. 10.1007/s11033-012-2286-4 23187733

[B21] Falcone Ferreyra M. L. Rius S. P. Casati P. (2012). Flavonoids: biosynthesis, biological functions, and biotechnological applications. Front. Plant Sci. 3:222. 10.3389/fpls.2012.00222 23060891 PMC3460232

[B22] Farmer E. E. Alméras E. Krishnamurthy V. (2003). Jasmonates and related oxylipins in plant responses to pathogenesis and herbivory. Curr. Opin. Plant Biol. 6, 372–378. 10.1016/S1369-5266(03)00045-1 12873533

[B23] Farrell J. D. Byrne S. Paina C. Asp T. (2014). *De novo* assembly of the perennial ryegrass transcriptome using an rna-seq strategy. PLoS ONE 9:e103567. 10.1371/journal.pone.0103567 25126744 PMC4134189

[B24] Gális I. Šimek P. Narisawa T. Sasaki M. Horiguchi T. Fukuda H.. (2006). A novel R2R3 MYB transcription factor NtMYBJS1 is a methyl jasmonate-dependent regulator of phenylpropanoid-conjugate biosynthesis in tobacco. Plant J. 46, 573–592. 10.1111/j.1365-313X.2006.02719.x 16640595

[B25] Ge Q. Zhang Y. Hua W.-P. Wu Y.-C. Jin X.-X. Song S.-H.. (2015). Combination of transcriptomic and metabolomic analyses reveals a JAZ repressor in the jasmonate signaling pathway of *Salvia miltiorrhiza*. Sci. Rep. 5. 10.1038/srep14048 26388160 PMC4585666

[B26] Grabherr M. G. Haas B. J. Yassour M. Levin J. Z. Thompson D. A. Amit I.. (2011). Full-length transcriptome assembly from RNA-Seq data without a reference genome. Nat. Biotechnol. 29, 644–652. 10.1038/nbt.1883 21572440 PMC3571712

[B27] Gupta A. K. Kaur N. (2005). Sugar signalling and gene expression in relation to carbohydrate metabolism under abiotic stresses in plants. J. Biosci. 30, 761–776. 10.1007/BF02703574 16388148

[B28] Heijari J. Nerg A. M. Kainulainen P. Viiri H. Vuorinen M. Holopainen J. K. (2005). Application of methyl jasmonate reduces growth but increases chemical defence and resistance against *Hylobius abietis* in Scots pine seedlings. Entomol. Exp. Appl. 115, 117–124. 10.1111/j.1570-7458.2005.00263.x

[B29] Hudson M. Ringli C. Boylan M. T. Quail P. H. (1999). The FAR1 locus encodes a novel nuclear protein specific to phytochrome A signaling. Genes Dev. 13, 2017–2027. 10.1101/gad.13.15.2017 10444599 PMC316922

[B30] Ismail I. Gor M.-C. Mohamed-Hussein Z.-A. Zainal Z. Noor N. M. (2011). Alteration of abiotic stress responsive genes in Polygonum minus roots by jasmonic acid elicitation, in Plants and Environment, eds Hemanth K. N. Vasanthaiah Kambiranda D. (Rijeka: InTech). 10.5772/23397

[B31] Kanehisa M. Goto S. (2000). KEGG: kyoto encyclopedia of genes and genomes. Nucleic Acids Res. 28, 27–30. 10.1093/nar/28.1.27 10592173 PMC102409

[B32] Kang S.-M. Jung H.-Y. Kang Y.-M. Yun D.-J. Bahk J.-D. Yang J.-K. (2004). Effects of methyl jasmonate and salicylic acid on the production of tropane alkaloids and the expression of PMT and H6H in adventitious root cultures of *Scopolia parviflora*. Plant Sci. 166, 745–751. 10.1016/j.plantsci.2003.11.022

[B33] Kazan K. Manners J. M. (2008). Jasmonate signaling: toward an integrated view. Plant Physiol. 146, 1459–1468. 10.1104/pp.107.115717 18390489 PMC2287326

[B34] Khairudin K. Sukiran N. A. Goh H.-H. Baharum S. N. Noor N. M. (2014). Direct discrimination of different plant populations and study on temperature effects by Fourier transform infrared spectroscopy. Metabolomics 10, 203–211. 10.1007/s11306-013-0570-5

[B35] Ku K. M. Juvik J. A. (2013). Environmental stress and methyl jasmonate-mediated changes in flavonoid concentrations and antioxidant activity in Broccoli florets and kale leaf tissues. Hortscience 48, 996–1002.

[B36] Less H. Galili G. (2008). Principal transcriptional programs regulating plant amino acid metabolism in response to abiotic stresses. Plant Physiol. 147, 316–330. 10.1104/pp.108.115733 18375600 PMC2330312

[B37] Li B. Dewey C. N. (2011). RSEM: accurate transcript quantification from RNA-Seq data with or without a reference genome. BMC Bioinform. 12:323. 10.1186/1471-2105-12-323 21816040 PMC3163565

[B38] Lindemose S. O'Shea C. Jensen M. K. Skriver K. (2013). Structure, function and networks of transcription factors involved in abiotic stress responses. Int. J. Mol. Sci. 14, 5842–5878. 10.3390/ijms14035842 23485989 PMC3634440

[B39] Liu J. Osbourn A. Ma P. (2015). MYB transcription factors as regulators of phenylpropanoid metabolism in plants. Mol. Plant 8, 689–708. 10.1016/j.molp.2015.03.012 25840349

[B40] Loke K.-K. Rahnamaie-Tajadod R. Yeoh C.-C. Goh H.-H. Mohamed-Hussein Z.-A. Noor N. M.. (2016). RNA-seq analysis for secondary metabolite pathway gene discovery in *Polygonum minus*. Genomics Data 7, 12–13. 10.1016/j.gdata.2015.11.003 26981350 PMC4778588

[B41] Lopez-Gomez R. Gomez-Lim M. (1992). A method for extracting intact RNA from fruits rich in polysaccharides using ripe mango mesocarp. HortScience 27, 440–442.

[B42] Maere S. Heymans K. Kuiper M. (2005). BiNGO: a cytoscape plugin to assess overrepresentation of gene ontology categories in biological networks. Bioinformatics 21, 3448–3449. 10.1093/bioinformatics/bti551 15972284

[B43] Magrane M. Consortium U. (2011). UniProt Knowledgebase: a hub of integrated protein data. Database 2011:bar009. 10.1093/database/bar009 21447597 PMC3070428

[B44] Maizura M. Aminah A. Wan Aida W. (2011). Total phenolic content and antioxidant activity of kesum (*Polygonum minus*), ginger (*Zingiber officinale*) and turmeric (*Curcuma longa*) extract. Int. Food Res. J. 18, 529.

[B45] Mandal S. (2015). Induction of phenolics, lignin and key defense enzymes in eggplant (*Solanum melongena* L.) roots in response to elicitors. Afr. J. Biotechnol. 9, 8038–8047. 10.5897/AJB10.984

[B46] Maucher H. Hause B. Feussner I. Ziegler J. Wasternack C. (2000). Allene oxide synthases of barley (*Hordeum vulgare* cv. Salome): tissue specific regulation in seedling development. Plant J. 21, 199–213. 10.1046/j.1365-313x.2000.00669.x 10743660

[B47] Md-Mustafa N. D. Khalid N. Gao H. Peng Z. Alimin M. F. Bujang N.. (2014). Transcriptome profiling shows gene regulation patterns in a flavonoid pathway in response to exogenous phenylalanine in *Boesenbergia rotunda* cell culture. BMC Genomics 15:984. 10.1186/1471-2164-15-984 25407215 PMC4289260

[B48] Mehrtens F. Kranz H. Bednarek P. Weisshaar B. (2005). The Arabidopsis transcription factor MYB12 is a flavonol-specific regulator of phenylpropanoid biosynthesis. Plant Physiol. 138, 1083–1096. 10.1104/pp.104.058032 15923334 PMC1150422

[B49] Memelink J. Verpoorte R. Kijne J. W. (2001). ORCAnization of jasmonate-responsive gene expression in alkaloid metabolism. Trends Plant Sci. 6, 212–219. 10.1016/S1360-1385(01)01924-0 11335174

[B50] Miao Z. Xu W. Li D. Hu X. Liu J. Zhang R.. (2015). *De novo* transcriptome analysis of *Medicago falcata* reveals novel insights about the mechanisms underlying abiotic stress-responsive pathway. BMC Genomics 16:818. 10.1186/s12864-015-2019-x 26481731 PMC4615886

[B51] Misra R. C. Maiti P. Chanotiya C. S. Shanker K. Ghosh S. (2014). Methyl jasmonate-elicited transcriptional responses and pentacyclic triterpene biosynthesis in sweet basil. Plant Physiol. 164, 1028–1044. 10.1104/pp.113.232884 24367017 PMC3912077

[B52] Moreira X. Sampedro L. Zas R. (2009). Defensive responses of Pinus pinaster seedlings to exogenous application of methyl jasmonate: concentration effect and systemic response. Environ. Exp. Bot. 67, 94–100. 10.1016/j.envexpbot.2009.05.015

[B53] Nabity P. D. Zavala J. A. DeLucia E. H. (2012). Herbivore induction of jasmonic acid and chemical defences reduce photosynthesis in *Nicotiana attenuata*. J. Exp. Bot. *ers*364. 64, 685–694. 10.1093/jxb/ers364 23264519 PMC3542056

[B54] Noir S. Bömer M. Takahashi N. Ishida T. Tsui T.-L. Balbi V.. (2013). Jasmonate controls leaf growth by repressing cell proliferation and the onset of endoreduplication while maintaining a potential stand-by mode. Plant Physiol. 161, 1930–1951. 10.1104/pp.113.214908 23439917 PMC3613466

[B55] Pérez-Clemente R. M. Vives V. Zandalinas S. I. López-Climent M. F. Mu-oz V. Gómez-Cadenas A. (2012). Biotechnological approaches to study plant responses to stress. BioMed Res. Int. 2013. 10.1155/2013/654120 23509757 PMC3591138

[B56] Pratelli R. Pilot G. (2014). Regulation of amino acid metabolic enzymes and transporters in plants. J. Exp. Bot. 65, 5535–5556. 10.1093/jxb/eru320 25114014

[B57] Qader S. W. Abdulla M. A. Chua L. S. Najim N. Zain M. M. Hamdan S. (2011). Antioxidant, total phenolic content and cytotoxicity evaluation of selected Malaysian plants. Molecules 16, 3433–3443. 10.3390/molecules16043433 21512451 PMC6260633

[B58] Ramakrishna A. Ravishankar G. A. (2011). Influence of abiotic stress signals on secondary metabolites in plants. Plant Signal. Behav. 6, 1720–1731. 10.4161/psb.6.11.17613 22041989 PMC3329344

[B59] Robinson M. D. McCarthy D. J. Smyth G. K. (2010). edgeR: a Bioconductor package for differential expression analysis of digital gene expression data. Bioinformatics 26, 139–140. 10.1093/bioinformatics/btp616 19910308 PMC2796818

[B60] Rojas C. M. Senthil-Kumar M. Tzin V. Mysore K. (2014). Regulation of primary plant metabolism during plant-pathogen interactions and its contribution to plant defense. Front. Plant Sci. 5:17. 10.3389/fpls.2014.00017 24575102 PMC3919437

[B61] Roslan N. D. Yusop J. M. Baharum S. N. Othman R. Mohamed-Hussein Z.-A. Ismail I.. (2012). Flavonoid biosynthesis genes putatively identified in the aromatic plant *Polygonum minus* via expressed sequences tag (EST) analysis. Int. J. Mol. Sci. 13, 2692–2706. 10.3390/ijms13032692 22489118 PMC3317681

[B62] Saibo N. J. Lourenço T. Oliveira M. M. (2009). Transcription factors and regulation of photosynthetic and related metabolism under environmental stresses. Ann. Bot. 103, 609–623. 10.1093/aob/mcn227 19010801 PMC2707349

[B63] Sasaki Y. Asamizu E. Shibata D. Nakamura Y. Kaneko T. Awai K.. (2001). Monitoring of methyl jasmonate-responsive genes in Arabidopsis by cDNA macroarray: self-activation of jasmonic acid biosynthesis and crosstalk with other phytohormone signaling pathways. DNA Res. 8, 153–161. 10.1093/dnares/8.4.153 11572481

[B64] Schluttenhofer C. Pattanaik S. Patra B. Yuan L. (2014). Analyses of *Catharanthus roseus* and *Arabidopsis thaliana* WRKY transcription factors reveal involvement in jasmonate signaling. BMC Genomics 15:502. 10.1186/1471-2164-15-502 24950738 PMC4099484

[B65] Schmittgen T. D. Livak K. J. (2008). Analyzing real-time PCR data by the comparative CT method. Nat. Protoc. 3, 1101–1108. 10.1038/nprot.2008.73 18546601

[B66] Shoji T. Hashimoto T. (2013). Jasmonate-responsive transcription factors: new tools for metabolic engineering and gene discovery, in Biotechnology for Medicinal Plants, eds. Chandra S. Lata H. Varma A. (Berlin: Springer), 345–357.

[B67] Tang W. Ji Q. Huang Y. Jiang Z. Bao M. Wang H.. (2013). FAR-RED ELONGATED HYPOCOTYL3 and FAR-RED IMPAIRED RESPONSE1 transcription factors integrate light and abscisic acid signaling in Arabidopsis. Plant Physiol. 163, 857–866. 10.1104/pp.113.224386 23946351 PMC3793063

[B68] Tatusov R. L. Fedorova N. D. Jackson J. D. Jacobs A. R. Kiryutin B. Koonin E. V.. (2003). The COG database: an updated version includes eukaryotes. BMC Bioinform. 4:41. 10.1186/1471-2105-4-41 12969510 PMC222959

[B69] Turner J. G. Ellis C. Devoto A. (2002). The jasmonate signal pathway. Plant Cell 14, S153–S164. 10.1105/tpc.000679 12045275 PMC151253

[B70] Van Moerkercke A. Galván-Ampudia C. S. Verdonk J. C. Haring M. A. Schuurink R. C. (2012). Regulators of floral fragrance production and their target genes in petunia are not exclusively active in the epidermal cells of petals. J. Exp. Bot. 63, 3157–3171. 10.1093/jxb/ers034 22345641 PMC3350925

[B71] Van Verk M. C. Gatz C. Linthorst H. J. (2009). Transcriptional regulation of plant defense responses. Adv. Bot. Res. 51, 397–438. 10.1016/S0065-2296(09)51010-5

[B72] Vikram P. Chiruvella K. K. Ripain I. H. A. Arifullah M. (2014). A recent review on phytochemical constituents and medicinal properties of kesum (*Polygonum minus* Huds.). Asian Pac. J. Trop. Biomed. 4, 430–435. 10.12980/APJTB.4.2014C1255 25182942 PMC3994350

[B73] Vom Endt D. Kijne J. W. Memelink J. (2002). Transcription factors controlling plant secondary metabolism: what regulates the regulators? Phytochemistry 61, 107–114. 10.1016/S0031-9422(02)00185-1 12169302

[B74] Wang H. Wang H. (2015). Multifaceted roles of FHY3 and FAR1 in light signaling and beyond. Trends Plant Sci. 20, 453–461. 10.1016/j.tplants.2015.04.003 25956482

[B75] Wang Y. Pan Y. Liu Z. Zhu X. Zhai L. Xu L.. (2013). *De novo* transcriptome sequencing of radish (*Raphanus sativus* L.) and analysis of major genes involved in glucosinolate metabolism. BMC Genomics 14:585. 10.3389/fpls.2016.00585 24279309 PMC4046679

[B76] Wasternack C. Hause B. (2013). Jasmonates: biosynthesis, perception, signal transduction and action in plant stress response, growth and development. An update to the 2007 review in Annals of Botany. Ann. Bot. 111, 1021–1058. 10.1093/aob/mct067 23558912 PMC3662512

[B77] Wenping H. Yuan Z. Jie S. Lijun Z. Zhezhi W. (2011). *De novo* transcriptome sequencing in *Salvia miltiorrhiza* to identify genes involved in the biosynthesis of active ingredients. Genomics 98, 272–279. 10.1016/j.ygeno.2011.03.012 21473906

[B78] Wu Z.-J. Li X.-H. Liu Z.-W. Xu Z.-S. Zhuang J. (2014). *De novo* assembly and transcriptome characterization: novel insights into catechins biosynthesis in *Camellia sinensis*. BMC Plant Biol. 14:277. 10.1186/s12870-014-0277-4 25316555 PMC4203915

[B79] Xie C. Mao X. Huang J. Ding Y. Wu J. Dong S.. (2011). KOBAS 2.0: a web server for annotation and identification of enriched pathways and diseases. Nucleic Acids Res. 39, W316–W322. 10.1093/nar/gkr483 21715386 PMC3125809

[B80] Xu Z. Zhang C. Zhang X. Liu C. Wu Z. Yang Z.. (2013). Transcriptome profiling reveals auxin and cytokinin regulating somatic embryogenesis in different sister lines of cotton cultivar CCRI24. J. Integr. Plant Biol. 55, 631–642. 10.1111/jipb.12073 23710882

[B81] Yaacob K. B. (1990). Essential oil of *Polygonum minus* Huds. J. Essent. Oil Res. 2, 167–172. 10.1080/10412905.1990.9697855

[B82] Yang C. Q. Fang X. Wu X. M. Mao Y. B. Wang L. J. Chen X. Y. (2012a). Transcriptional regulation of plant secondary metabolism. J. Integr. Plant Biol. 54, 703–712. 10.1111/j.1744-7909.2012.01161.x 22947222

[B83] Yang D. Du X. Yang Z. Liang Z. Guo Z. Liu Y. (2014). Transcriptomics, proteomics, and metabolomics to reveal mechanisms underlying plant secondary metabolism. Eng. Life Sci. 14, 456–466. 10.1002/elsc.201300075

[B84] Yang D.-L. Yao J. Mei C.-S. Tong X.-H. Zeng L.-J. Li Q.. (2012b). Plant hormone jasmonate prioritizes defense over growth by interfering with gibberellin signaling cascade. Proc. Natl. Acad. Sci. U.S.A. 109, E1192–E1200. 10.1073/pnas.1201616109 22529386 PMC3358897

[B85] Ye J. Fang L. Zheng H. Zhang Y. Chen J. Zhang Z.. (2006). WEGO: a web tool for plotting GO annotations. Nucleic Acids Res. 34, W293–W297. 10.1093/nar/gkl031 16845012 PMC1538768

[B86] Yu Z.-X. Li J.-X. Yang C.-Q. Hu W.-L. Wang L.-J. Chen X.-Y. (2012). The jasmonate-responsive AP2/ERF transcription factors AaERF1 and AaERF2 positively regulate artemisinin biosynthesis in *Artemisia annua* L. Mol. Plant 5, 353–365. 10.1093/mp/ssr087 22104293

[B87] Zhao J. Davis L. C. Verpoorte R. (2005). Elicitor signal transduction leading to production of plant secondary metabolites. Biotechnol. Adv. 23, 283–333. 10.1016/j.biotechadv.2005.01.003 15848039

[B88] Zhao S. Tuan P. A. Li X. Kim Y. B. Kim H. Park C. G.. (2013). Identification of phenylpropanoid biosynthetic genes and phenylpropanoid accumulation by transcriptome analysis of *Lycium chinense*. BMC Genomics 14:802. 10.1186/1471-2164-14-802 24252158 PMC4046672

