# Supplementary material for: Differential Gene Expression Analysis in Polygonum minus Leaf upon 24 h of Methyl Jasmonate Elicitation
Source: Front Plant Sci. 2017 Feb 6;8:109. doi: 10.3389/fpls.2017.00109 (PMC5292430; doi:10.3389/fpls.2017.00109)
Supplement: Supplementary file 1 [file Table1.PDF]

Supplementary Table 1 Primer sequences used in the qRT-PCR

Category	ID	Primer	Sequence (5'->3')
**Phenylpropanoid Biosynthesis**	C4H	Forward	ACAAGAGGCGCAGAGGAGGA
		Reverse	CTATCACCGCCGTTCCGGTC
	4CL	Forward	AGCCTGATAACGAGCGTGGC
		Reverse	CTTGTGGACGAGCAGGACGG
	PAL	Forward	TTGCAGCCCCAACTACCTGC
		Reverse	CTTCCTCCACCGCCTTTGGG
	CFI	Forward	TGGCGGTGTGCTTGATTTGC
		Reverse	ACACCAAGCCACTTCCTCGAC
	CHS	Forward	CAAGCTGGGGCTCAAGGAGG
		Reverse	GCGCTCGACATGTTCCCGTA
	DFR	Forward	CCGTCCGTGACCCTACCAAC
		Reverse	AAGTGGGTGTTCGCCGTAGG
	F3H	Forward	TCCACCATGGAAACCCACCC
		Reverse	CGGGTGACTCGGAAATGGGA
**JA Complex**	COI	Forward	GTCCCTCACGATCATTTGCCTG
		Reverse	CGCCGAGTTGCTGAGGATTG
	MYC	Forward	GCCGACCCGGAATGGTTCTA
		Reverse	TTGTAGTGGCGGAGGGTGTG
	JAZ	Forward	CCGTTGATTGTGGCTGATGGC
		Reverse	AGCTGGCATGGGAACAGGTC
**Transcription factor**	NAC	Forward	CCAGATTGCGGTGGTTTGGG
		Reverse	TCCTCGGCACAAAGCTCCAA
	MYB	Forward	ACATCGGAGCATTGGGCTGT
		Reverse	GAGGCTTCAGGCTACCAACCA
	ERF	Forward	TCCGCCCATTCTCAGGGTTG
		Reverse	CGCCGGGGATAAGACATGGA
	RAP	Forward	CGCAGGGTGATAGAGGGGAGG
		Reverse	AATTGAGCAGCCACAAAGGAACAA
	WRKY	Forward	CTGAAACCCCCTGCTCCACC
		Reverse	TGAGACCAGTAGCGGCACAC
**JA Biosynthesis**	PLA	Forward	TGACGCAGCTCGAATGGCTC
		Reverse	GGAGAGCTTGCAGTACGGGC
	PLD	Forward	TTCCTCCCTGTTTGCGAGGC
		Reverse	GCAAGGGATTGGTTCGCAGC
	MFP	Forward	CCGGGATCTGCATCGCATCG
		Reverse	GCGCTTCCGCCATTTGACTG
	OPR	Forward	TCATCCCGACTCTCCCTGCT
		Reverse	AGCTGGATTCGATGGGGTGG
	AOC	Forward	TAAGGCGGCAGAGGGAGGAC
		Reverse	CAAACCCTTGGCCACCTCCG
	AOS	Forward	TCGAGCCGGTCCTTGATGGG
		Reverse	CTGCCCTCCCTCAGCTCCTC
	LOX	Forward	ATACTTCCCGAACCGGCCCA
		Reverse	TCGCTTGGATTTGCGAGGGG
**Reference**	CDPK	Forward	ACGGAACCTTTCAGACCCGT
		Reverse	GCACCAGTTGGAAGAGCGTG
