# Supplementary material for: Differential Gene Expression Analysis in Polygonum minus Leaf upon 24 h of Methyl Jasmonate Elicitation
Source: Front Plant Sci. 2017 Feb 6;8:109. doi: 10.3389/fpls.2017.00109 (PMC5292430; doi:10.3389/fpls.2017.00109)
Supplement: Supplementary file 2 [file Table2.PDF]

Supplementary Table 2 Statistics of RNA-Seq data before and after read filtering (Sequencing platform: Illumina, Sequencing type: Paired-end, Minimum read size: 30 bp)

Sample	Read size (bp)	Library size (bp)	GC (%)	Details	Before pre-processing	After Processing		
						Total reads	Paired reads	Orphan reads
**Control-a**	90	200	48.06	**Total number of read**	36,508,536	36,485,556	36,463,352	22,204
				**Total read size**	3,285,758,240	3,271,250,424	3,269,266,111	1,984,313
**Control-b**	90	200	49.43	**Total number of read**	44,735,676	44,587,317	44,503,742	83,575
				**Total read size**	4,026,210,840	4,002,142,488	3,994,680,431	7,462,057
**Treated-a**	90	200	48.99	**Total number of read**	55,471,894	55,401,980	55,363,344	38,636
				**Total read size**	4,992,470,460	4,971,196,901	4,967,747,344	3,449,517
**Treated-b**	90	200	48.96	**Total number of read**	55,451,866,	55,339,717	55,235,362	104,355
				**Total read size**	4,990,667,940	4,967,700,684	4,958,385,285	9,315,399
