# Supplementary material for: Differential Gene Expression Analysis in Polygonum minus Leaf upon 24 h of Methyl Jasmonate Elicitation
Source: Front Plant Sci. 2017 Feb 6;8:109. doi: 10.3389/fpls.2017.00109 (PMC5292430; doi:10.3389/fpls.2017.00109)
Supplement: Supplementary file 3 [file Table3.PDF]

Supplementary Table 3 Sequence statistics of *P. minus* leaf transcriptome assembly

**Pre-assembly**		**Post-assembly**	
Number of raw reads	192,167,972	Total unique transcripts	182,111
Number of processed reads	191,792,366	Total length (bases)	157,743,382
Total length (bases)	17,295,117,480	GC Percent	43.64
Average length (bases)	90	Average length (bases)	866
N50	90	N50	1,387
Size range (bases)	90	Size range (bases)	201-17,019
