# Supplementary material for: Differential Gene Expression Analysis in Polygonum minus Leaf upon 24 h of Methyl Jasmonate Elicitation
Source: Front Plant Sci. 2017 Feb 6;8:109. doi: 10.3389/fpls.2017.00109 (PMC5292430; doi:10.3389/fpls.2017.00109)
Supplement: Supplementary file 4 [file Table4.PDF]

Supplementary Table 4 KEGG-based pathways classification of DEGs with number of sequences in pathways (Path) and enzymes (Enzs)

No	Pathway	Seqs in Path	Enzs	Seqs in Enzs
**Amino acid metabolism**				
1	Alanine, aspartate and glutamate metabolism	3	2	3
2	Arginine and proline metabolism	4	3	4
3	Cysteine and methionine metabolism	3	2	3
4	Glycine, serine and threonine metabolism	3	3	3
5	Lysine biosynthesis	2	2	2
6	Lysine degradation	1	2	2
7	Phenylalanine metabolism	14	3	22
8	Phenylalanine, tyrosine and tryptophan biosynthesis	15	6	27
9	Tyrosine metabolism	1	1	1
10	Valine, leucine and isoleucine biosynthesis	2	2	2
11	Valine, leucine and isoleucine degradation	1	1	1
**Biosynthesis of other secondary metabolites**				
12	Aflatoxin biosynthesis	2	1	2
13	Flavonoid biosynthesis	9	3	5
14	Glucosinolate biosynthesis	1	2	5
15	Indole alkaloid biosynthesis	1	1	1
16	Phenylpropanoid biosynthesis	16	4	24
**Carbohydrate metabolism**				
17	Amino sugar and nucleotide sugar metabolism	4	2	4
18	Ascorbate and aldarate metabolism	2	2	2
19	Butanoate metabolism	1	1	1
20	C5-Branched dibasic acid metabolism	3	2	3
21	Citrate cycle (TCA cycle)	2	2	4
22	Fructose and mannose metabolism	10	4	10
23	Galactose metabolism	1	1	1
24	Glycolysis / Gluconeogenesis	12	4	12
25	Glyoxylate and dicarboxylate metabolism	8	4	8
26	Inositol phosphate metabolism	2	3	3
27	Pentose and glucuronate interconversions	7	4	7
28	Pentose phosphate pathway	14	6	14
29	Propanoate metabolism	5	3	5
30	Pyruvate metabolism	5	2	5
31	Starch and sucrose metabolism	13	7	13
**Energy metabolism**				
32	Carbon fixation in photosynthetic organisms	14	5	14

33	Carbon fixation pathways in prokaryotes	4	3	6
34	Methane metabolism	11	5	11
35	Nitrogen metabolism	7	2	7
36	Oxidative phosphorylation	2	1	2
37	Sulfur metabolism	2	1	2
**Global and overview maps**				
38	Biosynthesis of antibiotics	49	26	64
**Glycan biosynthesis and metabolism**				
39	Lipopolysaccharide biosynthesis	1	1	1
40	N-Glycan biosynthesis	3	2	3
41	Other glycan degradation	2	1	2
42	Various types of N-glycan biosynthesis	3	2	3
**Lipid metabolism**				
43	alpha-Linolenic acid metabolism	4	2	4
44	Biosynthesis of unsaturated fatty acids	2	1	2
45	Ether lipid metabolism	2	1	2
46	Fatty acid biosynthesis	2	1	2
47	Fatty acid degradation	2	1	2
48	Glycerolipid metabolism	2	1	2
49	Glycerophospholipid metabolism	3	2	3
50	Linoleic acid metabolism	2	1	2
51	Sphingolipid metabolism	4	2	4
52	Steroid hormone biosynthesis	4	2	4
**Metabolism of cofactors and vitamins**				
53	Folate biosynthesis	1	1	1
54	Nicotinate and nicotinamide metabolism	1	1	1
55	One carbon pool by folate	2	2	2
56	Pantothenate and CoA biosynthesis	2	2	2
57	Porphyrin and chlorophyll metabolism	5	3	5
58	Retinol metabolism	1	1	1
59	Riboflavin metabolism	1	1	1
60	Thiamine metabolism	2	2	2
61	Vitamin B6 metabolism	2	1	2
**Metabolism of other amino acids**				
62	beta-Alanine metabolism	1	1	1
63	Cyanoamino acid metabolism	3	2	3
64	Glutathione metabolism	3	1	3
65	Selenocompound metabolism	1	1	1
66	Taurine and hypotaurine metabolism	1	1	1

**Metabolism of terpenoids and polyketides**				
67	Diterpenoid biosynthesis	1	1	1
68	Terpenoid backbone biosynthesis	1	1	1
69	Tetracycline biosynthesis	2	1	2
70	Zeatin biosynthesis	1	1	1
**Nucleotide metabolism**				
71	Purine metabolism	18	6	18
72	Pyrimidine metabolism	9	4	9
**Xenobiotics biodegradation and metabolism**				
73	Aminobenzoate degradation	2	1	2
74	Chloroalkane and chloroalkene degradation	1	1	1
75	Chlorocyclohexane and chlorobenzene degradation	1	1	1
76	Drug metabolism - cytochrome P450	1	1	1
77	Drug metabolism - other enzymes	3	2	3
78	Metabolism of xenobiotics by cytochrome P450	1	1	1
79	Steroid degradation	3	1	3
80	Styrene degradation	1	1	1
**Translation**				
81	Aminoacyl-tRNA biosynthesis	2	1	2
**Signal transduction**				
82	Phosphatidylinositol signaling system	1	1	1
**Total**		**366**	**193**	**415**
