# Supplementary material for: Differential Gene Expression Analysis in Polygonum minus Leaf upon 24 h of Methyl Jasmonate Elicitation
Source: Front Plant Sci. 2017 Feb 6;8:109. doi: 10.3389/fpls.2017.00109 (PMC5292430; doi:10.3389/fpls.2017.00109)
Supplement: Supplementary file 5 [file Table5.PDF]

Supplementary Table 5 KEGG-based pathways classification of up- and down-regulated DEGs

Pathway	Up-Regulated Seqs in path	Down-Regulated Seqs in path
**Amino acid metabolism**		
Phenylalanine, tyrosine and tryptophan biosynthesis	15	0
Cysteine and methionine metabolism	3	0
Arginine and proline metabolism	2	2
Lysine degradation	1	0
Alanine, aspartate and glutamate metabolism	1	2
Lysine biosynthesis	1	1
Tyrosine metabolism	1	0
Valine, leucine and isoleucine biosynthesis	1	1
Valine, leucine and isoleucine degradation	1	0
Glycine, serine and threonine metabolism	0	3
Phenylalanine metabolism	13	1
**Biosynthesis of other secondary metabolites**		
Phenylpropanoid biosynthesis	13	3
Flavonoid biosynthesis	8	1
Aflatoxin biosynthesis	2	0
Glucosinolate biosynthesis	1	0
Indole alkaloid biosynthesis	0	1
**Carbohydrate metabolism**		
Fructose and mannose metabolism	1	9
Glycolysis / Gluconeogenesis	3	9
Pentose phosphate pathway	5	9
Starch and sucrose metabolism	5	8
Glyoxylate and dicarboxylate metabolism	1	7
Pentose and glucuronate interconversions	3	4
Amino sugar and nucleotide sugar metabolism	2	2
Ascorbate and aldarate metabolism	1	1
Butanoate metabolism	0	1
C5-Branched dibasic acid metabolism	2	1
Galactose metabolism	0	1
Inositol phosphate metabolism	1	1
Propanoate metabolism	4	1
Pyruvate metabolism	4	1
Citrate cycle (TCA cycle)	2	0
**Energy metabolism**		
Carbon fixation in photosynthetic organisms	1	13

Methane metabolism	2	9
Nitrogen metabolism	1	6
Oxidative phosphorylation	1	1
Carbon fixation pathways in prokaryotes	4	0
Sulfur metabolism	2	0
**Global and overview maps**		
Biosynthesis of antibiotics	32	17
**Glycan biosynthesis and metabolism**		
Other glycan degradation	2	0
Lipopolysaccharide biosynthesis	1	0
N-Glycan biosynthesis	1	2
Various types of N-glycan biosynthesis	1	2
**Lipid metabolism**		
alpha-Linolenic acid metabolism	4	0
Sphingolipid metabolism	3	1
Biosynthesis of unsaturated fatty acids	2	0
Fatty acid biosynthesis	2	0
Fatty acid degradation	2	0
Linoleic acid metabolism	2	0
Ether lipid metabolism	1	1
Glycerolipid metabolism	1	1
Glycerophospholipid metabolism	1	2
Steroid hormone biosynthesis	1	3
**Metabolism of cofactors and vitamins**		
Porphyrin and chlorophyll metabolism	1	4
One carbon pool by folate	0	2
Pantothenate and CoA biosynthesis	1	1
Retinol metabolism	0	1
Riboflavin metabolism	0	1
Thiamine metabolism	1	1
Vitamin B6 metabolism	1	1
Folate biosynthesis	1	0
Nicotinate and nicotinamide metabolism	1	0
**Metabolism of other amino acids**		
Glutathione metabolism	3	0
Selenocompound metabolism	1	0
Taurine and hypotaurine metabolism	1	0
beta-Alanine metabolism	0	1
Cyanoamino acid metabolism	0	3

**Metabolism of terpenoids and polyketides**		
Tetracycline biosynthesis	2	0
Terpenoid backbone biosynthesis	1	0
Diterpenoid biosynthesis	0	1
Zeatin biosynthesis	0	1
**Nucleotide metabolism**		
Purine metabolism	11	7
Pyrimidine metabolism	5	4
**Xenobiotics biodegradation and metabolism**		
Steroid degradation	1	2
Aminobenzoate degradation	0	2
Chloroalkane and chloroalkene degradation	0	1
Chlorocyclohexane and chlorobenzene degradation	0	1
Drug metabolism - cytochrome P450	0	1
Drug metabolism - other enzymes	2	1
Metabolism of xenobiotics by cytochrome P450	0	1
Styrene degradation	1	0
**Translation**		
Aminoacyl-tRNA biosynthesis	0	2
**Signal transduction**		
Phosphatidylinositol signaling system	0	1
