# Supplementary material for: Differential Gene Expression Analysis in Polygonum minus Leaf upon 24 h of Methyl Jasmonate Elicitation
Source: Front Plant Sci. 2017 Feb 6;8:109. doi: 10.3389/fpls.2017.00109 (PMC5292430; doi:10.3389/fpls.2017.00109)
Supplement: Supplementary file 6 [file Table6.PDF]

**Supplementary Table 6** Represented differentially expressed TFs in MeJA-elicited *P. minus* leaf

| No | TF family | DEGs | Up regulated | Down regulated | No | TF family | DEGs | Up regulated | Down regulated |
|---|---|---|---|---|---|---|---|---|---|
| **1** | FAR1 | 56 | 22 | 34 | **28** | GeBP | 4 | 1 | 3 |
| **2** | C3H | 39 | 10 | 29 | **29** | mTERF | 4 | 4 | 0 |
| **3** | MYB-related | 25 | 17 | 8 | **30** | SNF2 | 4 | 4 | 0 |
| **4** | AP2-EREBP | 23 | 14 | 9 | **31** | AUX/IAA | 3 | 2 | 1 |
| **5** | NAC | 23 | 13 | 10 | **32** | C2C2-Dof | 3 | 2 | 1 |
| **6** | MADS | 22 | 12 | 10 | **33** | Coactivator p15 | 3 | 2 | 1 |
| **7** | MYB | 21 | 19 | 2 | **34** | LOB | 3 | 3 | 0 |
| **8** | SET | 21 | 12 | 9 | **35** | SWI/SNF-BAF60b | 3 | 3 | 0 |
| **9** | bHLH | 17 | 5 | 12 | **36** | ARID | 2 | 0 | 2 |
| **10** | ABI3VP1 | 15 | 10 | 5 | **37** | BSD | 2 | 2 | 0 |
| **11** | C2H2 | 15 | 8 | 7 | **38** | CAMTA | 2 | 0 | 2 |
| **12** | Orphans | 14 | 6 | 8 | **39** | CCAAT | 2 | 1 | 1 |
| **13** | SBP | 14 | 8 | 6 | **40** | CPP | 2 | 2 | 0 |
| **14** | TRAF | 14 | 8 | 6 | **41** | RB | 2 | 0 | 2 |
| **15** | bZIP | 13 | 7 | 6 | **42** | SWI/SNF-SWI3 | 2 | 0 | 2 |
| **16** | GNAT | 12 | 5 | 7 | **43** | TAZ | 2 | 2 | 0 |
| **17** | PHD | 11 | 8 | 3 | **44** | TCP | 2 | 1 | 1 |
| **18** | WRKY | 11 | 5 | 6 | **45** | Tify | 2 | 0 | 2 |
| **19** | GRAS | 8 | 4 | 4 | **46** | BES1 | 1 | 0 | 1 |
| **20** | HB | 7 | 6 | 1 | **47** | C2C2-CO-like | 1 | 0 | 1 |
| **21** | RWP-RK | 7 | 2 | 5 | **48** | C2C2-YABBY | 1 | 1 | 0 |
| **22** | FHA | 5 | 2 | 3 | **49** | E2F-DP | 1 | 1 | 0 |
| **23** | G2-like | 5 | 2 | 3 | **50** | GRF | 1 | 1 | 0 |
| **24** | HSF | 5 | 5 | 0 | **51** | HMG | 1 | 1 | 0 |
| **25** | Jumonji | 5 | 1 | 4 | **52** | LUG | 1 | 1 | 0 |
| **26** | C2C2-GATA | 4 | 1 | 3 | **53** | Pseudo ARR-B | 1 | 1 | 0 |
| **27** | DBP | 4 | 3 | 1 | **54** | VOZ | 1 | 1 | 0 |
