# Supplementary material for: Differential Gene Expression Analysis in Polygonum minus Leaf upon 24 h of Methyl Jasmonate Elicitation
Source: Front Plant Sci. 2017 Feb 6;8:109. doi: 10.3389/fpls.2017.00109 (PMC5292430; doi:10.3389/fpls.2017.00109)
Supplement: Supplementary file 10 [file Image1.PDF]

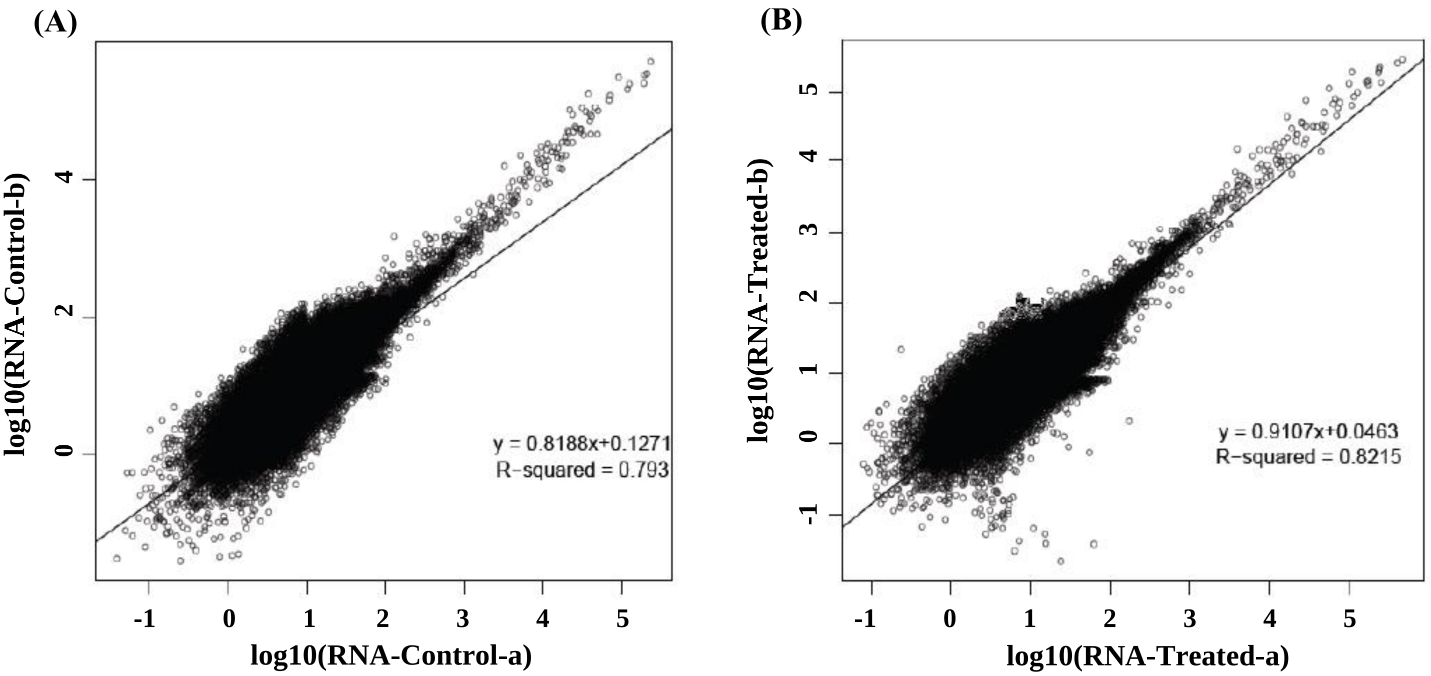

Supplementary Figure S1 (A) Correlation between RNA-Control replicates (FPKM > 0), (B) Correlation between MeJA-treated replicates (FPKM > 0).
