# Supplementary figures and images for: Differential Gene Expression Analysis in Polygonum minus Leaf upon 24 h of Methyl Jasmonate Elicitation
Source: Front Plant Sci. 2017 Feb 6;8:109. doi: 10.3389/fpls.2017.00109 (PMC5292430; doi:10.3389/fpls.2017.00109)

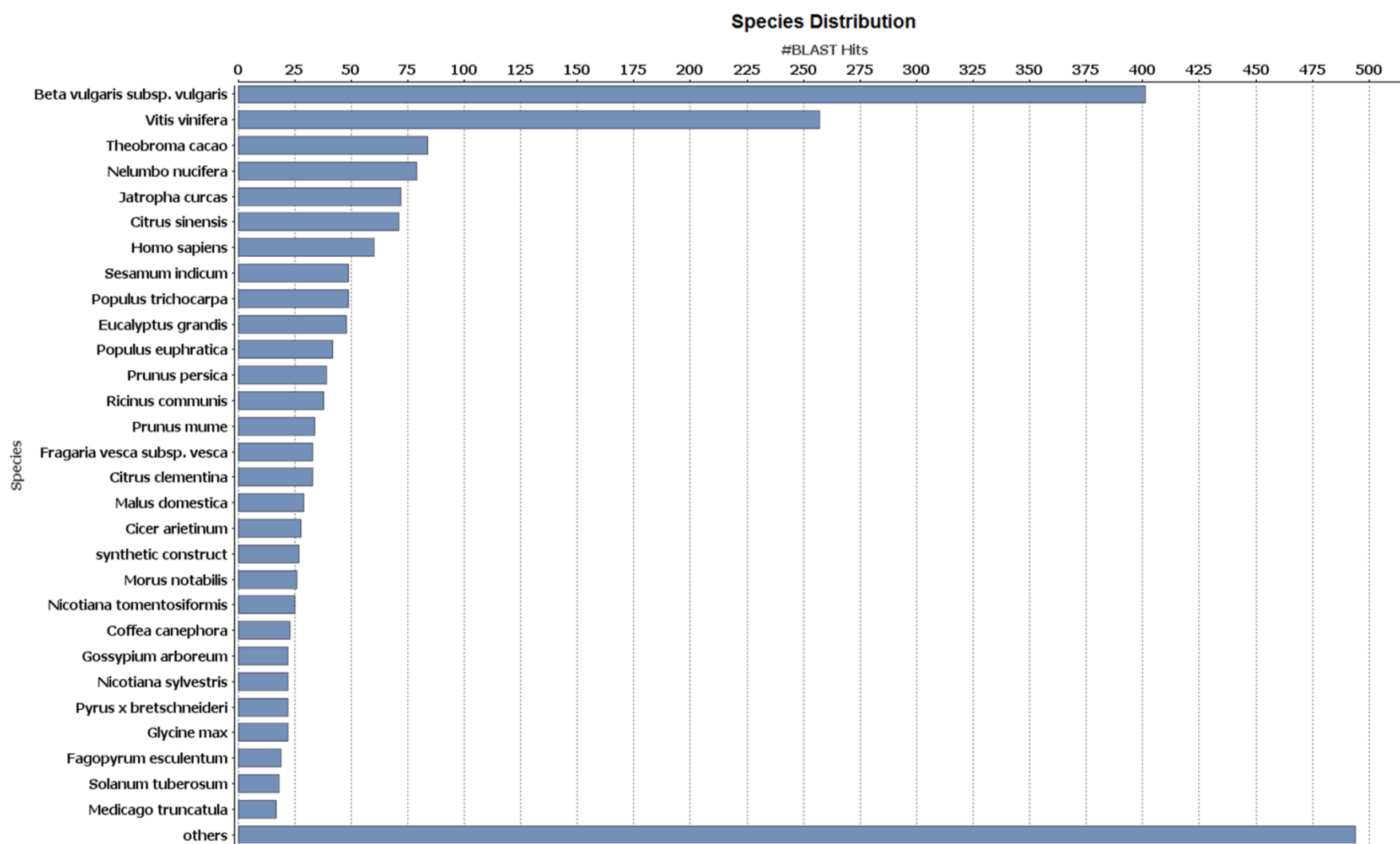

**Supplementary Figure S2** Distribution of top blast hit species from similarity search of DEG.

Supplement: Supplementary file 11 [file Image2.PDF]

**(A)**

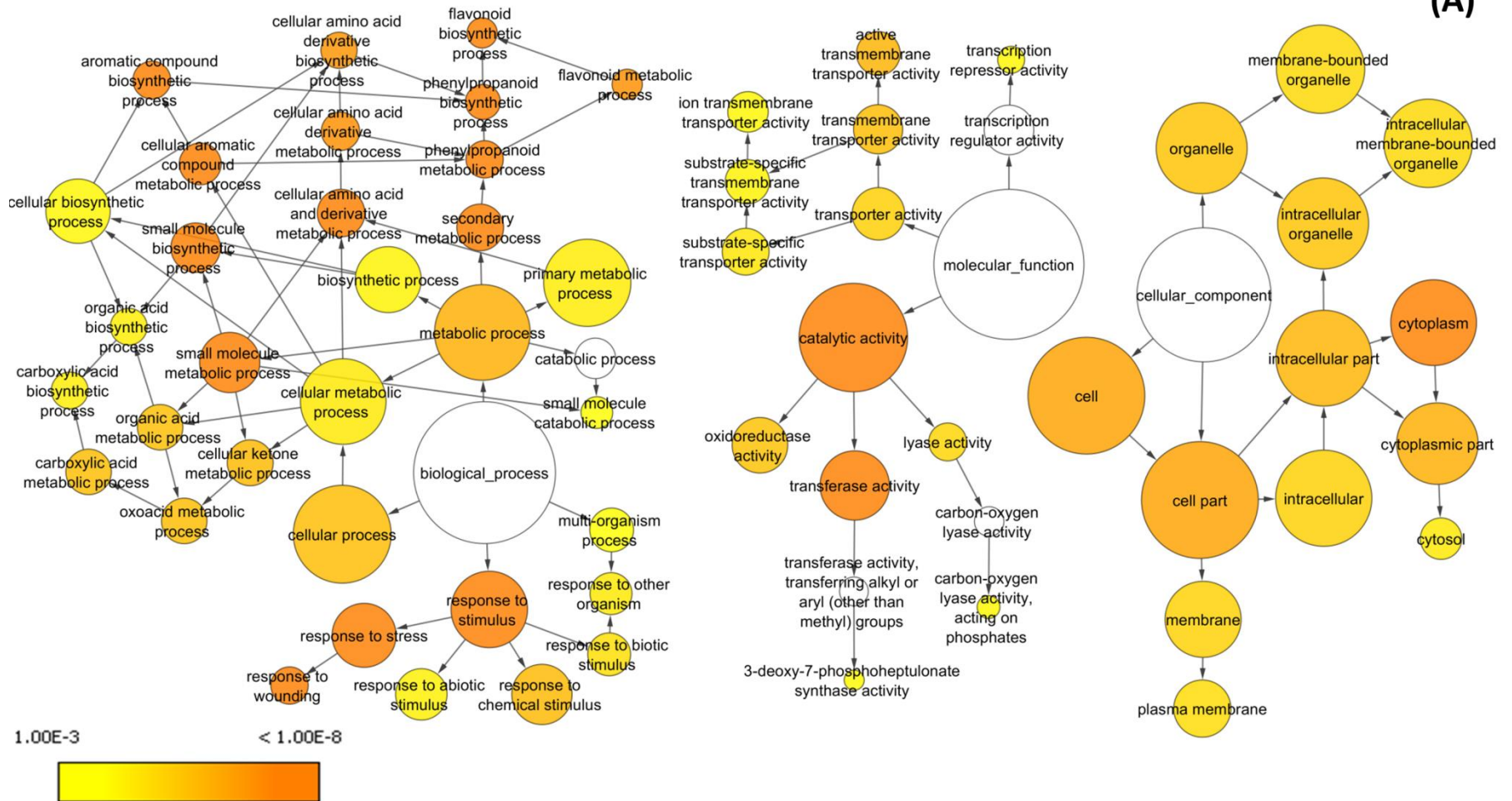

Supplement: Supplementary file 13 [file Image4.PDF]

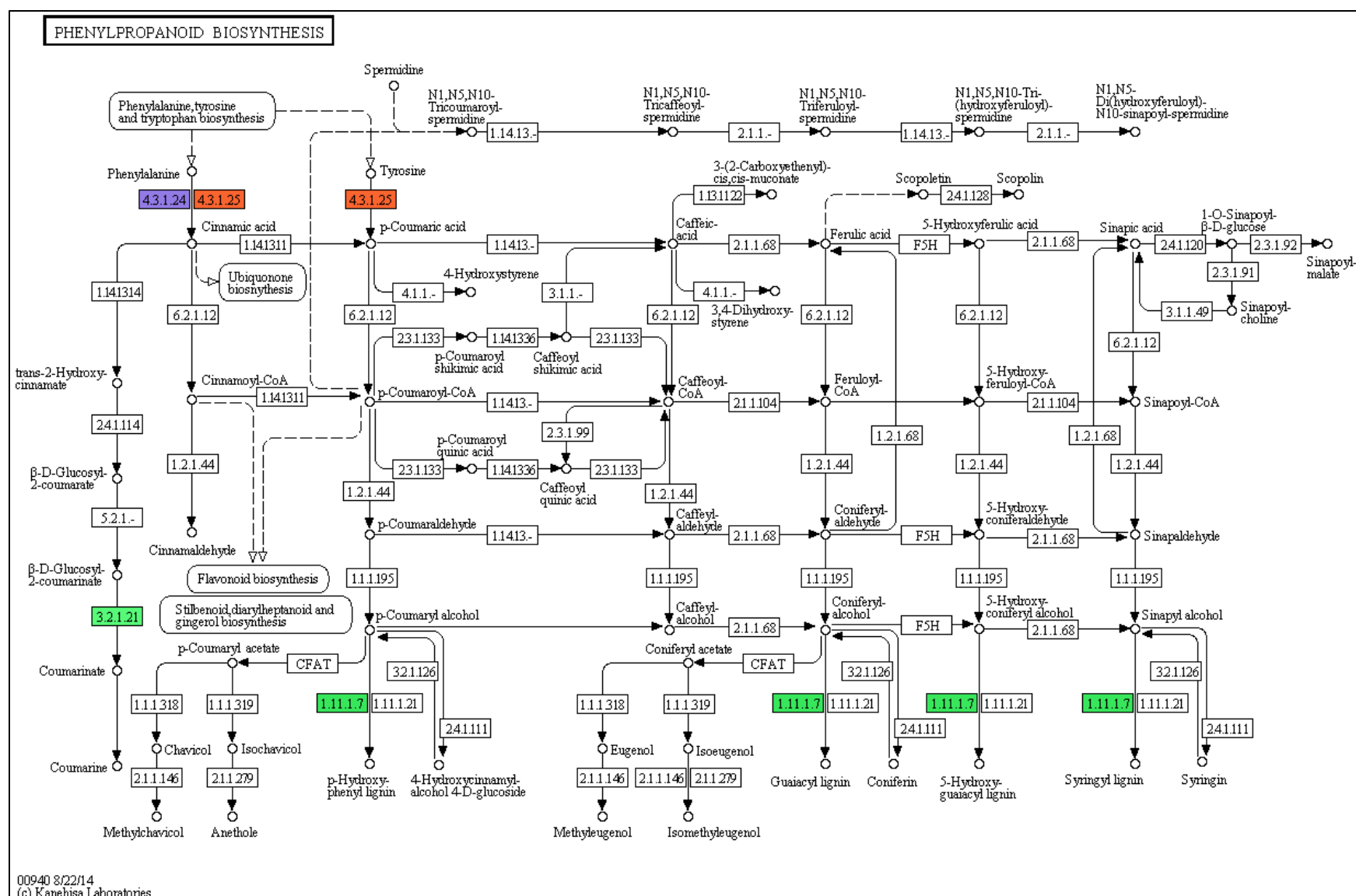

Supplement: Supplementary file 16 [file Image7.PDF]
