# Supplementary material for: Differential Gene Expression Analysis in Polygonum minus Leaf upon 24 h of Methyl Jasmonate Elicitation
Source: Front Plant Sci. 2017 Feb 6;8:109. doi: 10.3389/fpls.2017.00109 (PMC5292430; doi:10.3389/fpls.2017.00109)
Supplement: Supplementary file 12 [file Image3.PDF]

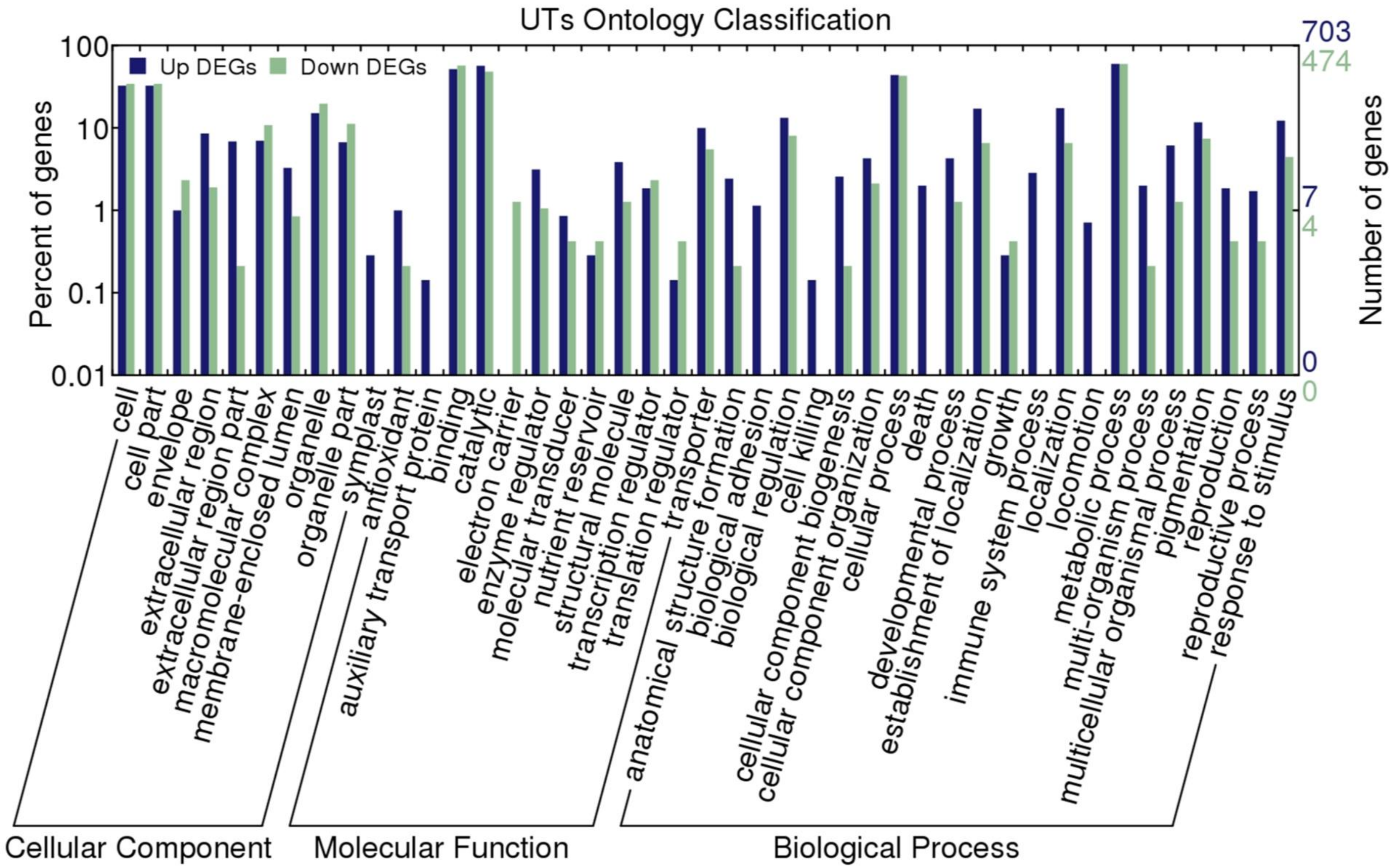

Supplementary Figure S3 Gene Ontology functional classification of up-regulated (Navy-blue) and down-regulated (Green) DEGS in *P. minus* leaf transcriptome. The results are summarised in three GO categories: biological process, molecular function and cellular component . The right y-axis indicates the number of DEGs in each category and the left y-axis indicates the percentage of sequences in the same category.
