# Supplementary material for: Differential Gene Expression Analysis in Polygonum minus Leaf upon 24 h of Methyl Jasmonate Elicitation
Source: Front Plant Sci. 2017 Feb 6;8:109. doi: 10.3389/fpls.2017.00109 (PMC5292430; doi:10.3389/fpls.2017.00109)
Supplement: Supplementary file 15 [file Image6.PDF]

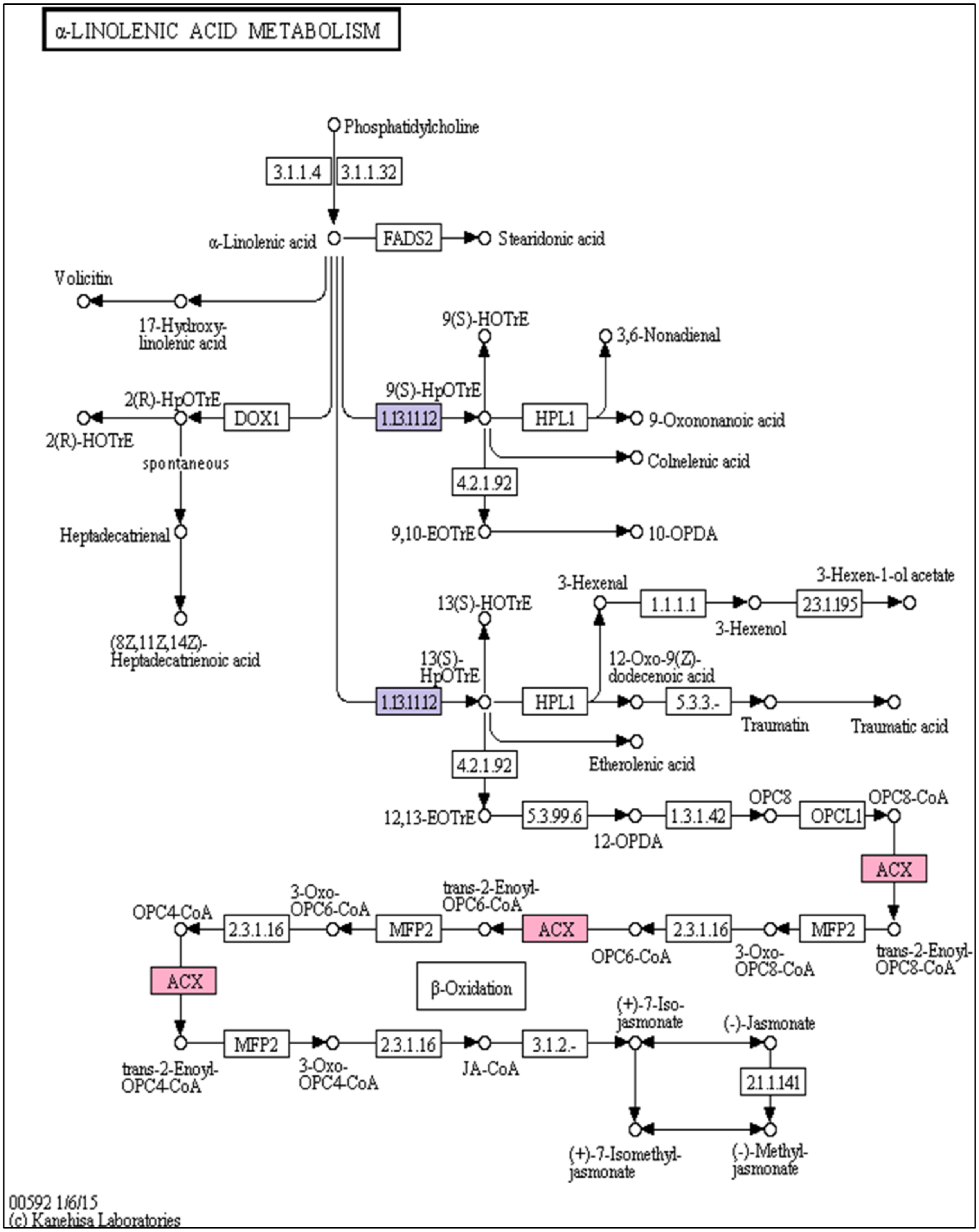

Supplementary Figure S6 KEGG pathway annotation analyses of DEGs in *P. minus* leaf. Alpha-Linolenic acid metabolism pathway (map00592) is one of the significant pathways detected after methyl jasmonate elicitation. Colored boxes indicate regulation upon MeJA.
