# Supplementary material for: Differential Gene Expression Analysis in Polygonum minus Leaf upon 24 h of Methyl Jasmonate Elicitation
Source: Front Plant Sci. 2017 Feb 6;8:109. doi: 10.3389/fpls.2017.00109 (PMC5292430; doi:10.3389/fpls.2017.00109)
Supplement: Supplementary file 17 [file Image8.PDF]

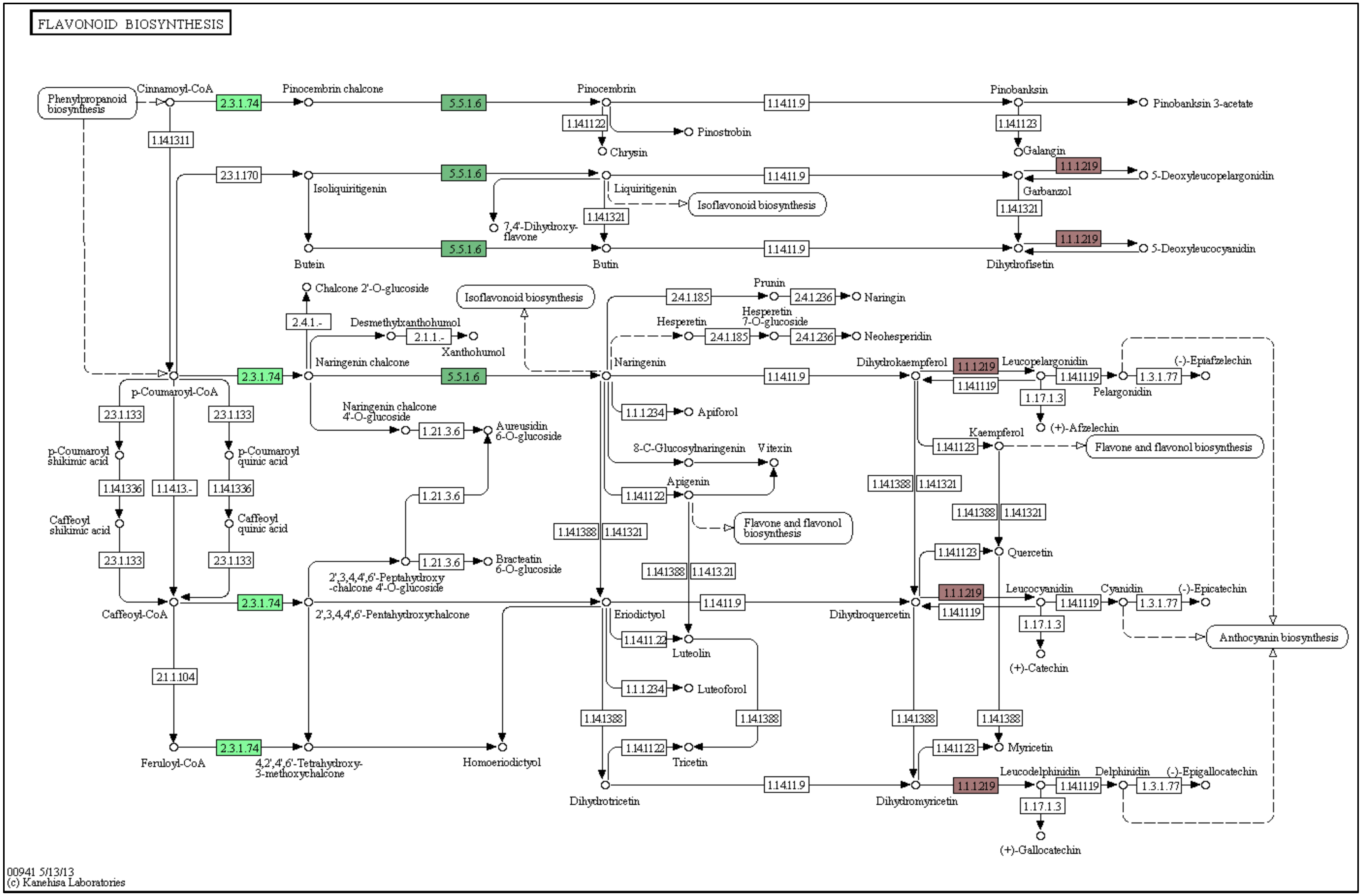

Supplementary Figure S8 KEGG pathway annotation analyses of DEGs. Flavonoid biosynthesis pathway (map00941) is one of the significant pathways detected after methyl jasmonate elicitation. Colored boxes indicate regulation upon MeJA.
